# Single-Atom Catalysts on Goldene

**DOI:** 10.1021/acscatal.5c01820

**Published:** 2025-06-13

**Authors:** Silvia Picello, Elisabetta Inico, Clara Saetta, Giovanni Di Liberto, Gianfranco Pacchioni

**Affiliations:** Department of Materials Science, 9305 University of Milano-Bicocca, Via Cozzi 55, 20125 Milano, Italy

**Keywords:** goldene, SAC, DFT, HER, OER

## Abstract

In single-atom catalysis, the interaction between isolated metal atoms and the supporting matrix plays a crucial role in determining the stability and reactivity of the system. This has driven the search for supporting materials, particularly two-dimensional (2D) materials, where graphene has been the predominant choice. Simultaneously, increasing attention is being given to single-atom alloys (SAAs), a subclass of single-atom catalysts (SACs), where the supporting matrix itself is a metal. Recently, Kashiwaya et al. [*Nature Synthesis* **3,** 744 (2024)] reported the synthesis of goldene, a 2D monolayer of Au(111) described as the gold analogue of graphene. Motivated by this breakthrough, we explored a class of SACs consisting of transition metal (TM) atoms stabilized on goldene. Through electronic structure calculations, we identified several systems that remain stable under both reducing and oxidizing conditions. We then investigated their catalytic performance in the hydrogen evolution reaction (HER) and oxygen evolution reaction (OER), discovering that certain TM-goldene systems exhibit promising activity, with reactivity significantly different from the same TMs supported on bulk Au(111). Our analysis included a comprehensive evaluation of potential reaction intermediates, extending beyond the conventional species typically assumed in HER and OER. This study provides strong theoretical evidence that SACs embedded in goldene could offer promising stability and catalytic reactivity.

## Graphical abstract

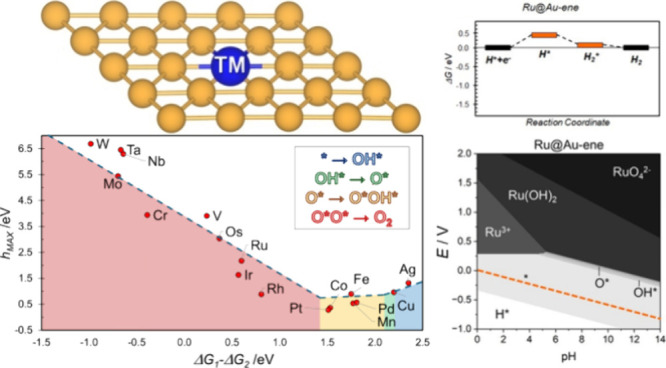

## Introduction

Single-atom catalysis is an emerging approach that bridges the gap between homogeneous and heterogeneous catalysis.^1–9^ A Single-Atom Catalyst (SAC) is a paradigm of single-site isolation,^10,11^ where metal atoms are atomically dispersed on a given support. This in principle leads to the maximization of the active phase, an important aspect to lower the loading of precious metals. Importantly, SACs share common features with organometallic compounds,^5,12,13^ where the properties and reactivity can be tuned by adjusting the local coordination environment.^14–16^ This opens the way to the potential rational design of new systems with desired activity toward specific reactions.^17–19^ The spectrum of potential applications of SACs is very broad, including electrochemical evolution of hydrogen and oxygen from liquid water,^20,21^ reduction of nitrogen to ammonia,^22,23^ CO_2_ to fuels,^24^ organic synthesis^25–32^ and others.^33^

In addition to the intrinsic properties of SACs, the nature of the supporting matrix is equally important as the active metal atom itself, due to its influence on the local coordination environment.^34,35^ This motivates the search for novel supporting matrices where SACs can be stabilized. The most commonly used supporting matrices are 2D materials, primarily carbon-based, such as graphene and carbon nitride.^36–38^ Many other systems are emerging, such as covalent organic frameworks,^39,40^ titanium nitride,^41,42^ and metal surfaces. In the latter case, a metal impurity in a metallic environment leads to the so-called Single-Atom Alloys (SAA).^43–48^

The search for new catalysts is driven by their potential to improve a wide range of chemical reactions. In this study, we focus on the Hydrogen Evolution Reaction (HER) and Oxygen Evolution Reaction (OER) due to their relatively simple mechanisms and critical role in water splitting. Nonetheless, emerging single-atom catalysts (SACs) also hold significant promise for other applications, such as the synthesis of fine chemicals and pharmaceutical compounds, where selectivity often outweighs activity. In such contexts, even catalysts that are less common or more challenging to synthesize may offer the distinct advantages necessary to make a process economically feasible.

Recently, Kashiwaya et al proposed the synthesis of a novel stable 2D material made by a single layer of Au(111), named goldene (Au-ene) in analogy with graphene.^49^ This system represents a new frontier in catalyst synthesis,^50^ as it unifies the concept of a 2D material, as graphene, with a metal surface, as gold.

Motivated by this breakthrough, we examined a set of SACs supported on goldene to evaluate their behavior in catalytic reactions. To the best of our knowledge, this is the first report of SACs supported on goldene. In selected cases, we compared the properties of SACs on goldene with those of the same atoms embedded on the (111) surface of metallic gold. The synthesis of goldene begins with Ti_3_SiC_2_, a well-known MAX phase material where ‘M’ is a transition metal (titanium), ‘A’ is a group A element (silicon), and ‘X’ is carbon. One can replace the silicon atoms with gold to create Ti_3_AuC_2_ layers within the structure. To isolate the gold layers, the Ti_3_AuC_2_ compound can be treated by wet chemical etching with reagents that selectively remove the titanium carbide layers, leaving the single-atom-thick gold sheets. Finally, to avoid agglomeration surfactant molecules are added. To produce the TM@Au-ene SACs discussed in this paper, a possible synthetic strategy could involve introducing trace amounts of the TM element during the initial substitution of Si in the MAX phase, the first step of goldene synthesis.

We first predicted the stability of 18 transition metals (TM) atoms in Au-ene in electrochemical conditions, Figure 1, using a recently proposed scheme developed by some of us.^51^ This is a crucial preliminary step when assessing the potential of SACs for electrochemical reactions. We considered all TMs except for the very early elements, Sc, Y, La, Ti, Zr, Hf, Tc (unstable) and Re to sample the representative behavior of TM elements. Subsequently, the SACs were tested for their activity in the Hydrogen Evolution Reaction (HER) and Oxygen Evolution Reaction (OER). The chemistry of these systems is more complex than usually assumed, and the entire set of possible intermediates has been considered for the two reactions, an essential aspect for the screening of SACs activity.^52,53^ For both HER and OER, several candidates exhibit low thermodynamic reaction barriers, comparable to those of the best noble metal species. Furthermore, the role of the supporting metal, whether a single layer of Au or bulk Au, significantly influences the reactivity, as notable differences are observed between the two systems. We hope that the findings of this study will inspire the experimental development of SACs based on goldene as novel catalytic systems.

**Figure 1:**
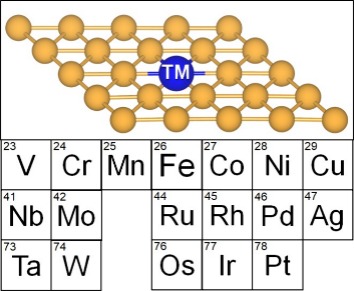
Structure of a single atom incorporated on goldene (TM@Au-ene) and the investigated TMs.

## Results

### Structure and Stability of TM@Au-ene

We begin with the full optimization of the Au monolayer (goldene), derived from the bulk crystal structure of Au along the (111) direction. The confinement of the system to two dimensions results in an overall lattice contraction of approximately 8%, in agreement with the findings of Kashiwaya et al.^49^ The calculated lattice parameters *a*, *b* and γ of the simulation cell are 16.304 Å, 16.304 Å and 120° respectively. In the original work, the measured Au-ene layer is contracted by about 9% with respect to bulk gold, with an Au-Au distance of 2.62 Å.^49^ In our calculations the lattice contraction in Au-ene is 8%, with the Au-Au distance passing from 2.94 Å (Au bulk) to 2.72 Å. In the same work the authors calculated with DFT the Au-Au distance and reported a value of 2.74 Å.^49^ The incorporation of metal impurities was achieved by substituting an Au atom with a transition metal (TM) dopant, a common approach in SAAs.^44,49,54^ After geometry relaxation, the dopant occupies a surface site without further structural changes. The atoms do not exhibit any significant protrusion. Table 1 reports the calculated binding energies, the number of unpaired electrons derived from spin density, and the distance between the TM atoms and their first Au neighbors and the spin density of the isolated metal atoms. The binding energy of the TMs was calculated with respect to the isolated metal atom. Another alternative choice is to calculate the stability with respect to the bulk phase.^55^ We decided to adopt the first criterion as the binding energy calculated in this way can be used as proxi to assess the stability of the metal atom in a matrix. Several TM atoms maintain an atomic magnetization similar to that of the free atom.^56^ It must be mentioned that the inherent assumption here is that metal atoms are quite distant, i.e. they tend to be isolated in the Au-ene matrix. Here, we took one case, Pt@Au-ene, and we performed a simple test calculation. We simulated two Pt atoms embedded in Au-ene at different distances. The most stable conditions is obtained when the two atoms are very distant, (*d*_Pt‑Pt_ = 16.30 Å), as when the distance is reduced to 8.15 Å, the structure is 0.38 eV less stable. Even when two atoms are nearest-neighbors, *d*_Pt‑Pt_ = 2.56 Å, the system is less stable by 0.13 eV. Of course, one should perform a more robust set of calculations to provide conclusive statements about this point.

**Table 1:** Calculated Binding Energy (*E*_b_), Number of Unpaired Electrons (*N_e_*), TM-Au Distance (*d*) for TM@Au-ene, Working *U* Parameter of TM, and the Spin Density of the Isolated Metal

TM@Au-ene	*E*_b_/eV	*N*_*e*_	*d*_TM‑Au_/Å	*U*/eV	spin density
V	–6.40	2.38	2.71	2.72	3.57
Cr	–5.26	3.83	2.68	3.79	4.66
Mn	–6.01	4.50	2.70	3.06	4.74
Fe	–6.05	3.60	2.68	3.29	3.88
Co	–6.22	2.62	2.68	3.42	2.93
Ni	–5.78	1.25	2.67	3.40	–0.66
Cu	–5.29	0.00	2.66	4.20	0.31
Nb	–7.43	1.54	2.71	2.02	3.10
Mo	–5.75	2.90	2.71	2.30	4.39
Ru	–6.44	3.15	2.70	2.79	2.57
Rh	–5.97	1.81	2.71	3.04	–0.01
Pd	–4.29	0.92	2.71	3.33	0.00
Ag	–4.60	0.00	2.71	3.57	0.34
Ta	–8.09	1.08	2.71	1.87	2.16
W	–7.58	2.39	2.71	2.08	3.12
Os	–6.20	3.69	2.70	2.51	3.99
Ir	–6.05	1.81	2.69	2.74	2.87
Pt	–6.73	0.47	2.71	2.95	0.00

An important consideration in the theoretical study of SACs for electrochemical applications is their stability under working pH and applied voltage conditions. This can be assessed using the formalism of Pourbaix diagrams (see Section S1).^57^ The stability diagrams are evaluated as a function of the pH-potential couples, where *E* denotes the applied potential with respect to the Standard Hydrogen Electrode (SHE). For each value of pH and voltage the most stable state for the catalyst is determined, including possible dissolution processes, formation of adsorbed species, etc. In this respect, a key quantity is the binding energy of the TM atom to the support, a proxy of the strength of the interaction.^55,58^ We restricted the analysis for the stability plot to *, H*, OH*, O*, OOH*. A representative example is reported in Figure 2a, Ag@Au-ene (the diagrams of all other SACs considered are reported in Figures S1–S6). Goldene is predicted to be stable under both HER and OER conditions, while the stability of TM@Au-ene catalysts is highly system-dependent, Figure 2b. Some metals, such as V, Cr, Nb, Mo, Ta, and Os, are prone to dissolution in both HER and OER conditions, at *E =* 0 *V* and *E =* 1.23 *V*, across both acidic and basic environments. These systems could exhibit excellent catalytic activities, but they would not survive in operating conditions. Other systems, such as Mn, Fe, Co, Ni, Cu, Ru, Rh, Pd, W and Ir, are stable in HER conditions, but unstable in strong oxidation conditions such as OER, as they tend to dissolve and oxidize. Only Ag and Pt are stable in both HER and OER conditions, irrespective of the pH.

**Figure 2:**
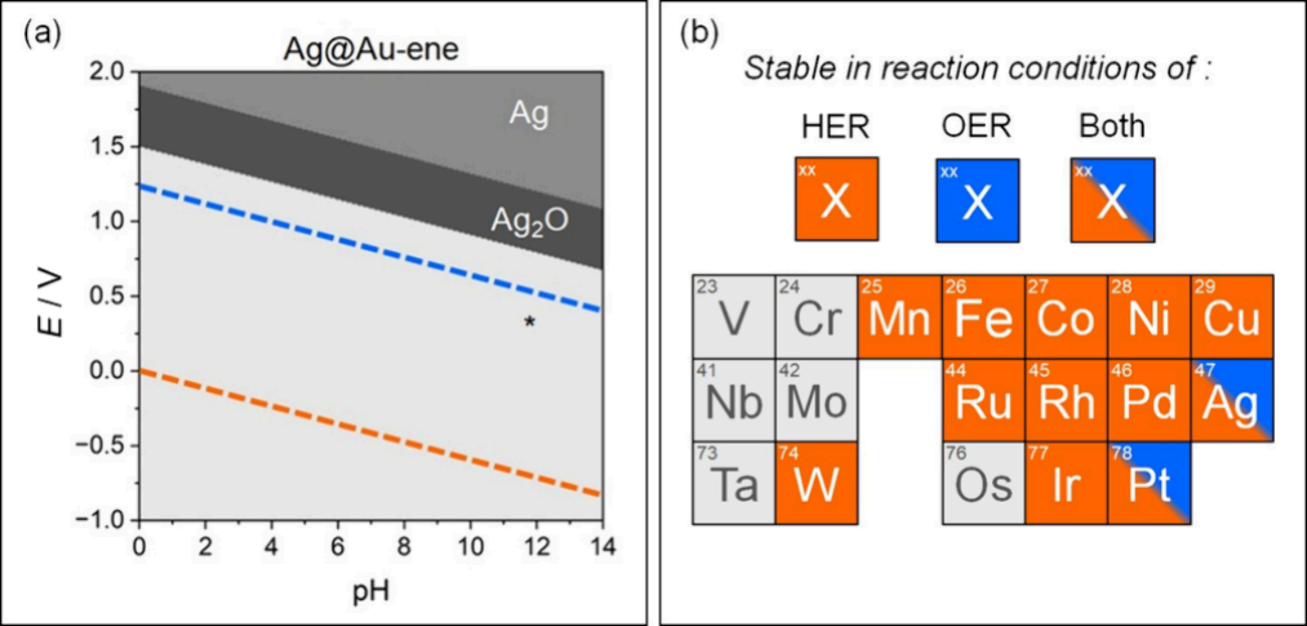
(a) Pourbaix diagram of Ag@Au-ene. The equilibrium potential curves of H_2_O/H_2_ (orange) and _2_/H_2_O (blue) couples are reported. Asterisk indicates the clean catalysts without adsorbed species. (b) Stability of the TM@Au-ene complexes. Atoms in orange or blue are stable in HER and/or OER conditions, respectively.

### TM@Au-ene in Hydrogen Evolution Reaction

Once investigated the structure and stability of TM@Au-ene, we test their reactivity in the HER. On extended metallic surfaces, the reaction occurs via the formation of a hydrogen adduct (H*) adsorbed on the catalyst *,^59,60^ Volmer step:

$$H^{+}+e^{-}+*\to H^{*}$$ 1

Then, the reaction can proceed via two distinct parallel mechanisms both releasing molecular H_2_. In one case, a second proton is reduced on the same site reacting with H*, Heyrovski step:

$$H^{+}+e^{-}+H^{*}\to H_{2}+*$$ 2

In the other case, two adsorbed H* adducts diffuse and combine and form H_2_, Tafel step:

$$H^{*}+H^{*}\to H_{2}+*$$ 3

All the simulated SACs can bind H, but their binding trends vary depending on the TM. Some metals, such as Cr, Ir, and Pt, are highly reactive, forming very stable adducts with Gibbs free energy of formation below −0.5 eV. In contrast, V, Mn, Fe, Co, Cu, and Ag are nearly inert, with Δ*G*_H_ values higher than 1 eV. Most metals (Ni, Nb, Mo, Ru, Rh, Pd, Ta, W, and Os) exhibit intermediate behavior. Notably, Nb, Ru, Rh, W, and Os show Δ*G*_H_ values close to zero, which is considered the ideal value for HER according to the Sabatier principle and the Computational Hydrogen Electrode (CHE) model (see Table S1).

However, in single-atom catalysis, it is not sufficient to consider only the free energy of H adsorption, H*, to predict HER reactivity.^53,55^ SACs are analogues of organometallic compounds and they can form complexes where two H atoms are bound to the same active site. This introduces an additional reaction intermediate besides the classical one (H*). Hydrogen complexes can be essentially of two types. The hydrogen molecule can be activated resulting in a H-H bond distance elongation, *d*_H‑H_ < 1.5 Å, as happens for V, Nb, Mo, Ru and Os. This is a Kubas-Crabtree-like hydrogen complex (H_2_*).^61–64^ Alternatively, the H-H bond can be completely broken, *d*_H‑H_ > 1.5 Å, leading to a dihydride complex (H*H*).^65,66^ This happens for Rh, Pd, Ta, W, Ir and Pt. A last possibility is that two hydrogen atoms are bound in equivalent positions of the lattice close to the TM (2H*), Figure S7a. Finally, a few metals (Cr, Mn, Fe, Co, and Ni) are unable to stably bind H_2_, resulting in a weakly physisorbed state.

Notice that in the vast majority of computational studies on this subject only adsorbed H atoms (H*) are considered for the HER, leading to potentially incorrect reaction paths. Table 2 reports the relevant structural information and formation Gibbs free energy of the adducts. Figure S8 reports all calculated Gibbs free energy profiles. The affinity of the catalyst for HER can be estimated by using as a proxy the maximum thermodynamic reaction barrier of the process, *h*_max_. Indeed, the maximum energy change depends both on Δ*G*_H*_ and Δ*G*_H2*_ leading to a three-dimensional volcano plot, Figure 3, and not to the classical two-dimensional volcano plot. Of course, the need to overcome two barriers rather than just one reduces the number of potential good candidates for the HER process, as shown in Figure 3. In particular, only W, Os, Nb, and Rh exhibit *h*_max_ values close to zero, which are compatible with good activity. All other complexes either bind one of the intermediates too strongly or too weakly. Finally, we underline that bare Au-ene is expected to be unreactive H_2_ is only physisorbed and the adsorption free energy of H* is very positive, Δ*G*_H*_ = 0.75 eV.

**Figure 3:**
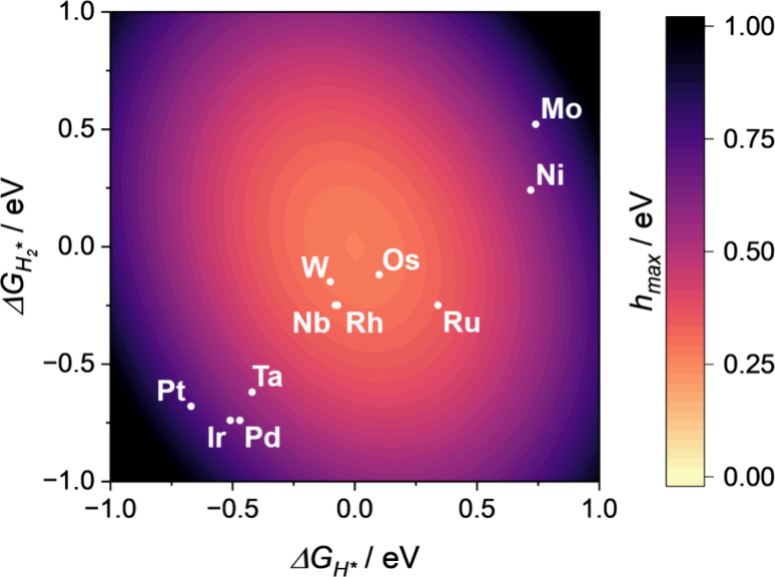
*h*_max_ is reported versus Δ*G*_H*_ and Δ*G*_H2*_ (formation of H* and H_2_* intermediates, respectively) resulting in a three-dimensional volcano plot for HER on 18 TM@Au-ene complexes. Yellow: *h*_max_ close to 0 eV, high activity; black: *h*_max_ close to 1 eV, low activity.

**Table 2:** Formation Free Energy of the H_2_-Adducts^a^, Distance between the Hydrogen Atoms and the Metal (*d*_TM‑H_), H-H Distance, Nature of the Adduct, and Maximum Thermodynamic Barrier (*h*_max_)­^b^

TM@Au-ene	Δ*G*_H2*_/eV	*d*_TM‑H_/Å	*d*_H‑H_/Å	Adduct	*h*_*max*_*/* eV
V	0.01	2.03	0.78	H_2_*	1.42
Cr	–1.63	2.69	0.76	H_2phys_	1.63
Mn	0.27	2.56	0.76	H_2phys_	1.49
Fe	0.28	2.20	0.77	H_2phys_	1.40
Co	0.25	2.59	0.76	H_2phys_	1.61
Ni	0.24	1.97	0.77	H_2phys_	0.72
Cu	0.13	1.74	3.12	2H*	1.10
Nb	–0.25	2.01	0.80	H_2_*	0.25
Mo	0.52	1.69	1.11	H_2_*	0.74
Ru	0.07	1.59	1.05	H_2_*	0.34
Rh	–0.25	1.54	1.83	H*H*	0.25
Pd	–0.74	1.61	2.38	H*H*	0.74
Ag	0.22	2.01	3.63	2H*	1.28
Ta	–0.62	1.75	1.81	H*H*	0.62
W	–0.15	1.68	1.57	H*H*	0.15
Os	–0.12	1.58	1.43	H_2_*	0.12
Ir	–0.74	1.54	1.72	H*H*	0.74
Pt	–0.68	1.59	2.37	H*H*	0.68
Au	–0.09	3.20	0.75	H_2phys_	0.75

a For Δ*G*_H*_ see Table S1.

b The two TM-H distances are always the same.

We have also calculated the *G*_MAX_(η) descriptor introduced by Exner^67,68^ and compared it with *h*_MAX_. *G*_MAX_(η) was calculated at the value of η = 0.3 V. Interestingly, the results are consistent with both descriptors, Figure S15.

Figure 4a shows the HER energy profile considering the formation of both H* and H_2_* intermediates for the case of Rh@Au-ene, showing that (a) the reaction occurs via formation of a dihydride intermediate and (b) that the largest thermodynamic barrier, 0.25 eV, is compatible with a considerable activity. Figure 4b summarizes the predicted affinity of all TM@Au-ene complexes for HER. By combining the information from the calculated Gibbs free energy profiles and the stability under HER conditions, Ru, Rh, and W emerge as promising systems for further investigation.

**Figure 4:**
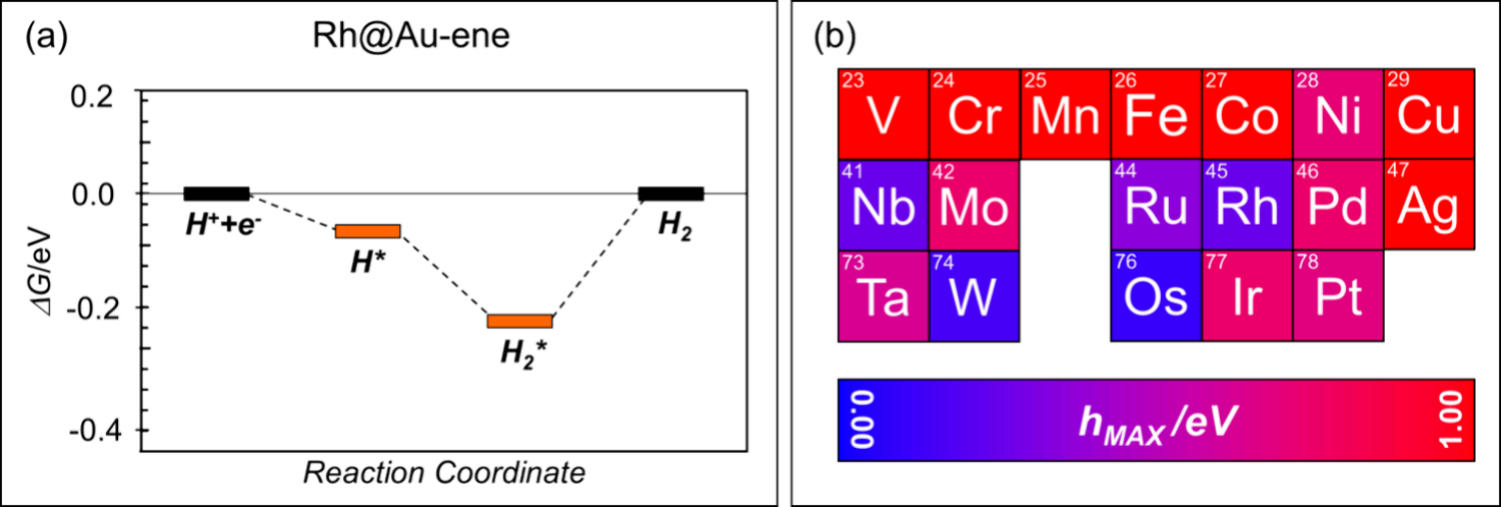
(a) Free energy diagram for the HER on Rh@Au-ene. (b) Activity of the TM atoms considered in this work. The reactivity of each atom is reported with a color scale, depending on the extent of the thermodynamic barrier (blue, *h*_max_ close to 0 eV; red, *h*_max_ > 1.00 eV).

### TM@Au-ene in Oxygen Evolution Reaction

Once investigated the cathodic part of water splitting, we move toward the evolution of molecular oxygen, which is most often the anodic part of electrochemical reduction processes of interests.^69–71^ The reaction exhibits a significant overpotential relative to the equilibrium potential. On conventional catalysts, it is believed to proceed via a four-electron transfer mechanism, involving the formation of three intermediates (OH*, O*, OOH*):^72^

$$*+H_{2}O\to {\mathrm{OH}}^{*}+H^{+}+e^{-}$$ 4

$${\mathrm{OH}}^{*}\to O^{*}+H^{+}+e^{-}$$ 5

$$O^{*}+H_{2}O\to {\mathrm{OOH}}^{*}+H^{+}+e^{-}$$ 6

$${\mathrm{OOH}}^{*}\to O_{2}+H^{+}+e^{-}+*$$ 7

In the case of SACs, it has been proposed that the reaction is more complex due to the formation of other adducts, whose formation energy is competitive with that of the classical ones. More specifically, after OH* forms, the system can adsorb a water molecule, leading to the formation of an OH*OH* intermediate, which is competitive with the formation of O*.^73,74^ All the species that can form are represented in Figure S7b.

$${\mathrm{OH}}^{*}+H_{2}O\to {\mathrm{OH}}^{*}{\mathrm{OH}}^{*}+H^{+}+e^{-}$$ 8

Similarly, the OOH* intermediate can compete with a O*OH* species.^74^

$${\mathrm{OH}}^{*}{\mathrm{OH}}^{*}\to O^{*}{\mathrm{OH}}^{*}+H^{+}+e^{-}$$ 9

$$O^{*}+H_{2}O\to O^{*}{\mathrm{OH}}^{*}+H^{+}+e^{-}$$ 10

Finally, before the release of molecular oxygen, an oxygen complex, peroxo or superoxo, can form (O_2_*).^74,75^ Oxygen complexes are classified based on the extent of O-O bond elongation. If the O-O bond experiences only a slight elongation from its equilibrium length in O_2_ (1.25 Å), the complex is identified as a superoxo species, 1.25 Å < *d*_OO_ < 1.35 Å,^76,77^ which typically assumes a η^1^ configuration. This happens for Fe, Rh, Pd, Ir, Pt (see Table S2). If instead, the O-O bond is further elongated, 1.35 Å < *d*_OO_ < 1.45 Å, the system is classified as a peroxo adduct with a η^2^ configuration.^76,78^ This occurs for V, Mo, Ru, Os. Another possibility is that the O-O bond is completely broken, forming a dioxo adduct (O*O*). This is the case for Cr, Mn, Co, Ni, Cu, Nb, Ag, Ta, and W (Table S2).

Figures S9–11 report the calculated reaction energy profiles for OER and Figure 5a shows a representative example, that of Pt@Au-ene. In all cases examined in this work, the most favorable reaction pathway involves the formation of at least one unconventional intermediate. The most favorable pathway is defined by the most stable intermediates. Similarly to HER, Au-ene binds too weakly the reaction intermediates of OER, Table 3.

**Figure 5:**
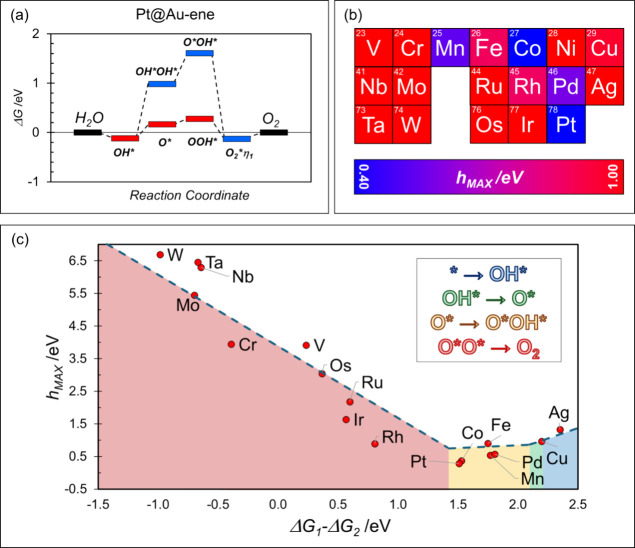
(a) Free energy diagram of OER on Pt@Au-ene, considering both classic (red) and unconventional (blue) intermediates; (b) activity of the TM atoms considered in this work. The reactivity of each atom is reported with a color scale, depending on the extent of the barrier (blue *h*_max_ < 0.40 eV, red *h*_max_ > 1.00 eV). (c) Calculated *h*_max_ for all the species presented in this work. Different colored areas correspond to the formation of different intermediates as indicated in the inset.

**Table 3:** Calculated Gibbs Free Energies, *h*_max_, for OER of TM@Au-ene at Two Potentials, *E* = 0 and 1.23 V

Δ*G* /eV	*E =* 0 *V*								*E* = 1.23 *V*
TM@Au-ene	OH*	O*	OOH*	OH*OH*	O*OH*	O*O*	**η**_**1**_ **O**_**2**_ *	**η**_**2**_ **O**_**2**_ *	*h*_max_*/* eV
V	–0.22	0.14		0.90	1.29	1.48			3.92
Cr	–0.31	–0.57	2.08	–0.11	0.92			1.46	3.94
Mn	1.34	3.23	4.64	3.21	4.28			5.39	0.54
Fe	0.93	2.80	4.42	2.77	3.67			4.47	0.90
Co	1.18	2.83	4.47	3.43	4.07		5.17		0.37
Ni	–0.17	3.12	4.80	3.81	4.37		5.86		1.94
Cu	2.09	4.42	5.13	4.23	5.47		5.50		0.97
Nb	–1.74	–2.25		–1.60	–1.52	–0.90			6.30
Mo	–0.23	–0.81		0.65	0.62	–0.05			5.45
Ru	0.67	1.39		2.17	3.20			3.22	2.18
Rh	1.06	2.00	4.16	3.05	4.38		4.50		0.89
Pd	1.23	3.16	4.26	3.76	5.11		4.94		0.58
Ag	2.68	5.15	5.20	4.72	5.89		6.71		1.33
Ta	–2.20	–2.75	–0.10	–2.39	–2.01	–1.06			6.46
W	–0.87	–1.73		–0.47	–0.86	–1.29			6.69
Os	0.49	0.98	3.88	1.82	2.72	2.36			3.04
Ir	0.79	1.48	3.87	2.67	3.82		3.76		1.64
Pt	1.23	2.87	4.25	3.61	5.05		5.24		0.28
Au	2.33	4.71	5.05	4.47	5.90				1.15

This further confirms that, for this class of SACs, limiting the analysis of the OER reaction pathway to the “classical” intermediates overlooks crucial reaction steps. This finding aligns with previous reports on SACs supported by carbon-based 2D materials.^74,79,80^

Table 3 reports all the calculated Gibbs free energies and the corresponding *h*_max_ values. Some systems, such as Co and Pt, display very small reaction energy differences calculated at the equilibrium potential of the OER reaction, *E* = 1.23 V.

Before closing this section, we discuss the possible existence of correlations between the stability of different intermediates in OER. On classical heterogeneous catalysts, universal (scaling) relations between the stability of the reaction intermediates have been established.^81^ In particular, the Gibbs free energy of formation of O* and of OOH* intermediates correlates with that of the OH* species.^82^ However, SACs often do not adhere to classical scaling relations.^83^ Nevertheless, we recently found that scaling relations can still be identified, provided that the formation of multiple distinct adducts is taken into account.^79^ This was obtained by using as a database a set of about 50 SACs supported on carbon-based matrices. Figure S12 reports the correlations between the stability of different intermediates and Δ*G*_OH_. From this it is clear that the O* intermediate is always more stable than the OH*OH* one, Figure S12a.^79^ OOH* and O*OH* are competitive species, and the most stable one depends on the capability of the SAC to bind OH*. SACs that strongly bind OH* lead to the O*OH* intermediate, Figure S12b. Finally, η^1^ and η^2^ O_2_ complexes are nearly isoenergetic, while O*O* species are often the most stable ones, Figure S12c. It must be mentioned that unconventional intermediates could form either in sin- and anticonfigurations. In this work we considered only the formation of sin adducts, under the assumption that only one side the surface is available to the reactants.

We considered a descriptor of OER, *x* = Δ*G*_2_ - Δ*G*_1_, as the free energy difference between the most stable intermediate involving the release of two electrons and the formation of the OH* species (which involves the release of one electron). Figure 5c shows that the maximum thermodynamic reaction barrier (*h*_max_) can be rationalized in a volcano-like plot depending on *x*. The lowest barriers are achieved when the catalyst binds the intermediates neither too strongly nor too weakly, with an optimal value around 1.4 eV, comparable to that of extended metal surfaces (*x* = 1.6 eV). The volcano plot features a left branch where the rate-determining step is the release of molecular O_2_ from the oxygen complex, as the catalyst binds the intermediates too strongly. On the right side, in the classical region, the catalyst binds the intermediates too weakly, making the formation of OH* the limiting step. The complex chemistry of SACs enables the formation of unconventional intermediates, giving rise to a new branch in the plot that is absent in classical heterogeneous catalysts. In this region, the rate-limiting steps involve alternative intermediate processes, such as OH* to O* or O* to O*OH*, corresponding to the area in Figure 5c with the lowest thermodynamic reaction barriers.

After evaluating the properties of SACs supported on Au-ene, their stability under electrochemical conditions, and their reactivity toward HER and OER, we summarize our findings in Figure 6. This Figure highlights systems that may be of interest for future dedicated studies on HER or OER. For instance, Ru, Rh, and W are stable under HER conditions and exhibit low barriers of 0.34 eV, 0.25 eV, and 0.15 eV, respectively. For OER, only Pt is predicted to be stable, with a low barrier of 0.28 eV. Thus, Pt@Au-ene is a system worthy of attention and further dedicated studies, as it is expected to be stable under OER conditions and to exhibit a low reaction barrier.

**Figure 6:**
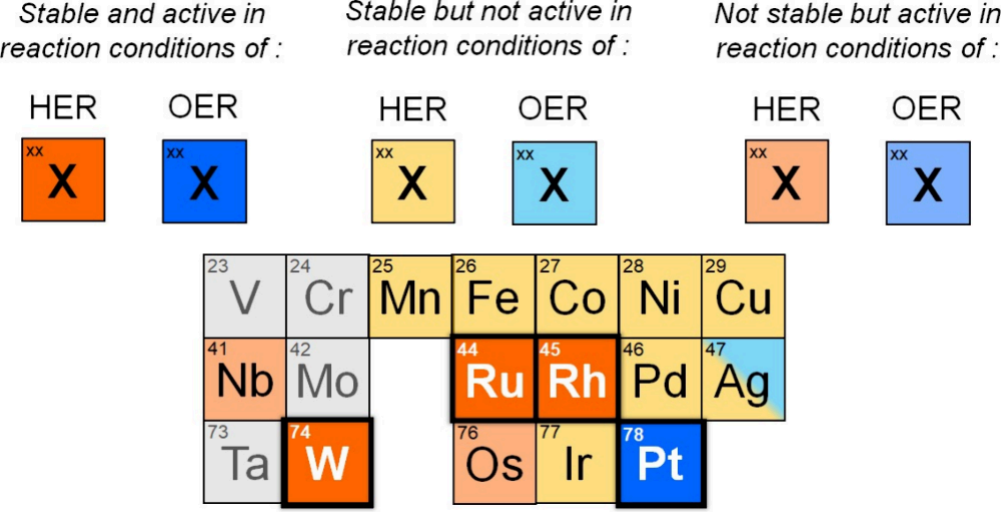
Table resuming stability and activity of the TM@Au-ene SACs presented in this work.

### TM@Au-ene Compared to TM@Au(111)

One might question whether using Au-ene provides a clear advantage over the conventional Au(111) surface in stabilizing isolated TM atoms and forming new SAAs. To address this, we examined a representative set of four systems, Ru, Rh, Pd, and Pt, and compared the reactivity of TM@Au-ene with that of TM@Au(111), where Au(111) refers to the (111) surface of bulk gold. To this end, an Au(111) slab was constructed with sufficient thickness (*d*) (five layers of Au, *d* ∼ 1 nm) and the corresponding SACs were tested in HER.^84^ The same DFT+*U* approach of TM@Au-ene was used to simulate TM@Au(111) catalysts to allow a direct comparison of the predictions.

Figure S13 compares the stability of SACs supported on Au-ene and Au(111). From this, it is evident that only minor differences in stability arise. However, while the stability of these systems is similar, their reactivity shows significant variation. As shown in Figure S13, the Gibbs free energy profiles reveal that the support has a significant impact on the properties of the active metal, resulting in both qualitative and quantitative changes. As a consequence, TM@Au-ene and TM@Au(111) show distinctly different reactivities. (See Table S3)

To provide an example, Figure 7a reports the case of Ru: the intermediates of the HER on Ru@Au-ene show Gibbs free energies close to zero eV, suggesting a good catalyst for HER, while Ru@Au(111) binds hydrogen too strongly, resulting in a poor HER catalyst. The case of Pt exhibits an opposite trend, since Pt@Au-ene binds too strongly the HER intermediates, resulting in low catalytic activity while Pt@Au(111) is an excellent catalyst with low thermodynamic reaction barriers, Figure 7b. There are also cases, such as Rh@Au-ene and Rh@Au(111), where no relevant differences in reactivity between the two supports are found, Figure S13b. On the contrary, Pd@Au-ene and Pd@Au(111) both exhibit high barriers, but for opposite reasons and due to a completely different behavior, Figure S13c. In particular, Pd@Au-ene binds the HER intermediates exergonically, whereas Pd@Au(111) binds them endergonically.

**Figure 7:**
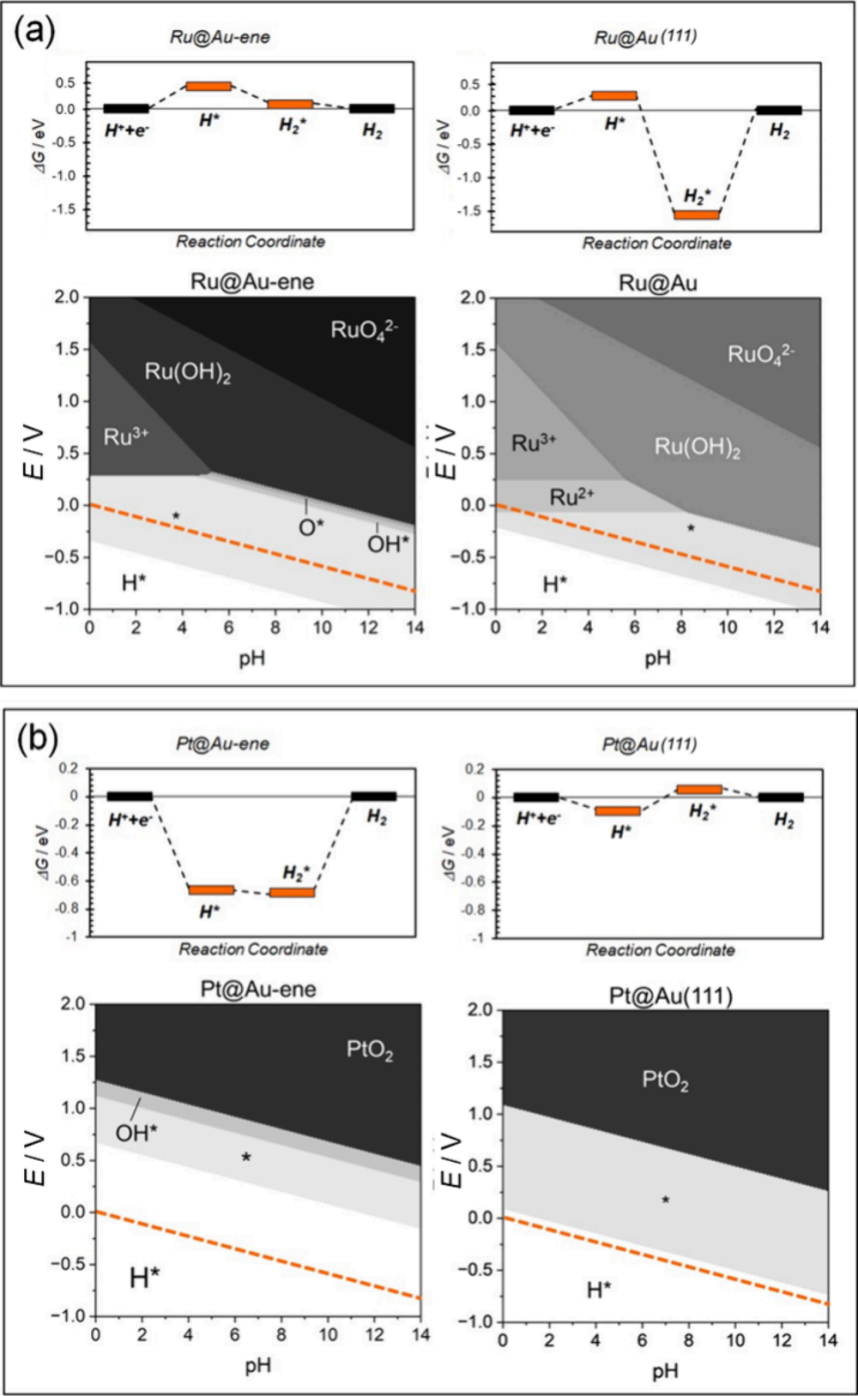
Reactivity and stability of SACs on Au-ene and Au(111). (a) Ru@Au-ene versus Ru@Au(111); (b) Pt@Au-ene versus Pt@Au(111). In the stability plots, the orange line represents the equilibrium potential of the H^+^/H_2_ couple. The most stable species at a given pH and potential condition are reported. The bare catalyst is indicated as asterisk. We restricted the analysis only to the classical intermediates.

These results clearly demonstrate that goldene exerts a strong influence on the chemistry of the trapped TM atom, likely due to its smaller lattice constants and different band structure. This further supports the idea that the surrounding environment is as important as the active site in determining the activity of SACs.

We have also considered the OER activity on the Pt@Au(111) catalyst. The results indicate a qualitative and quantitative difference in behavior of the catalysts. First, Figure S16 shows that Pt is active toward OER if embedded in Au-ene, while it becomes too reactive in Au(111) where some intermediates are too stable. Also, we notice that different reaction intermediates can form. On Au-ene, Pt prefers to form the conventional OER intermediates and a O_2_* adduct, while on Au(111) the reaction proceeds through other unconventional intermediates.

## Conclusions

We investigated the nature and reactivity of a series of single-atom catalysts (SACs) supported on Au-ene, the recently synthesized gold analogue of graphene, using density functional theory calculations. We considered a set of 18 transition metals embedded in Au-ene. Initially, we assessed the stability of these catalysts under electrochemical conditions, focusing on environments relevant to hydrogen evolution and oxygen evolution reactions. Our results show that the metal’s nature strongly influences stability, with certain candidates predicted to remain stable under both hydrogen and oxygen evolution conditions, such as Ag@Au-ene and Pt@Au-ene.

Next, we explored the reactivity of these SACs in hydrogen evolution, focusing on both the classical H* intermediate and including the formation of hydrogen complexes. We identified Rh, Ru, and W as particularly promising candidates. In the oxygen evolution reaction, we found that Pt stands out as a promising new catalyst. Also in this case, we consider either the classical OH*, O*, and OOH* intermediates and unconventional ones. In all cases, the most favorable reaction pathway involves the formation of at least one unconventional intermediate. Notably, the predicted catalytic activity can be explained through scaling relations, with the lowest reaction barriers around 0.4 eV at the OER equilibrium potential, comparable to state-of-the-art heterogeneous catalysts.

Overall, our findings underscore the potential of SACs supported on goldene and motivate future experimental efforts to realize their practical synthesis.

## Methods

### Computational Details

We performed quantum chemical calculations at the level of Density Functional Theory (DFT) as implemented in the Vienna Ab initio Simulation Package (VASP).^85–87^ The exchange-correlation functional was modeled by the Perdew-Burke-Ernzherov (PBE) parametrization.^88^ Dispersion forces were included by using the Grimme et al.’s D3 scheme.^89^ The valence electrons have been expanded on a set of plane waves with a kinetic cutoff of 400 eV, whereas core electrons were described by Projector Augmented Wave (PAW) pseudopotentials.^90,91^ We designed a working simulation cell with lattice vectors large to enough to have a small concentration of the dopant, *a* = *b* = 16.304 Å. In this case, given the large size of the simulation cell, we worked at Γ point. SACs can retain atomic magnetization due to their atomic-like character.^56^ For this reason, self-interaction corrected functionals must be adopted.^92,93^ Here, we used the DFT+*U* approach, with working *U* values of TM (Table 1) that have been already applied and tested in previous works, including SACs.^80,94–97^ The DFT+*U* method, which introduces a parametrized Hubbard *U* term, can sometimes yield spurious results depending on the specific choice of *U*. To mitigate this, the *U* values used in our study were not selected arbitrarily. Instead, they were adopted from a foundational work by Anisimov et al.^98^

We carefully benchmarked the performance of the DFT+*U* approach against more sophisticated functionals, such as the hybrid functionals PBE0 and HSE06.^92^ Our results show that PBE+*U*, when using the U values from the referenced study, produces results closely aligned with those of hybrid functionals and notably different from standard PBE results. However, this effect is primarily related to open-shell systems. In contrast, systems where the transition metal atom adopts a closed-shell ground state show far less sensitivity to the choice among PBE, PBE+*U*, or PBE0.

Nevertheless, it remains true that DFT results are inherently dependent on the chosen exchange-correlation functional. In the absence of experimental data for direct comparison, we acknowledge that there is no definitive way to determine the most accurate theoretical approach for these systems

We started from the bulk crystal structure of gold and we fully optimized the unit cell.^99^ The Au-ene model was created by optimizing an Au(111) monolayer and its lattice constants. Then, metal atoms were incorporated on the surface, replacing an Au atom, to model SACs.

The binding energy (*E*_b_) of the metal atoms to the support was calculated as:

$$E_{b}=E_{M@S}-E_{M}-E_{S}$$ 11

where *E*_M@S_, *E*_M_, and *E*_S_ are the energies of the TM@Au-ene, of the isolated metal atom, and of the Au-ene support where an Au vacancy has been created, respectively.

The free energies of the adsorbed species were calculated by means of the Computational Hydrogen Electrode (CHE) approach:^72,100,101^

$$\Delta G=\Delta E-T\Delta S+\Delta E_{\mathrm{ZPE}}$$ 12

where Δ*E* is the calculated DFT energy, *T*Δ*S* is the entropic contribution and Δ*E*_ZPE_ is the zero-point energy correction. The entropic contribution of adsorbates was calculated by using the formalism of the partition function.^102^ The entropy of isolated molecules was taken from the NIST database. The ZPE was calculated in a harmonic fashion. Section S5 reports all the details and the working equations. Table S4 reports the calculated entropic and zero-point energy contribution of all species considered in this work.

In this work we used the ab initio thermodynamic approach, i.e. we considered only thermodynamic barriers to extract information and trends on the reactivity of SACs, thus neglecting kinetic barriers.^100,101^

The stability of the SACs in electrochemical conditions was evaluated by means of the formalism of Pourbaix diagrams,^51^ taking insight from the seminal work by Norskov and co-workers.^58^ as discussed above and in Section S1.

The role of solvation was evaluated in a few selected cases to assess its importance. An accurate description of solvent effects includes long-range electrostatic interactions, hydrogen bonding, etc.^103–105^ Explicit solvation models, combined with ab initio molecular dynamics, are the best choice,^106,107^ but are computationally intensive, introduce configurational complexity, limiting the possibility of screening large sets of catalysts in different reactions. Implicit solvation models can provide a general indication of role of the solvent, although they may not fully capture the details of solute–solvent interactions.^107,108^ We tested the role of the solvent for a subset of SACs and we examined its effect on the HER for four representative examples, Rh@Au-ene, Ru@Au-ene, Pd@Au-ene and Pt@Au-ene. The implicit solvation model introduces a nearly systematic stabilization of the intermediates by about 0.15 eV, without changing the reactivity or the structural properties of the intermediates, Figure S14 and Table S5. For this reason, the following discussion is based on results obtained in absence of solvation. The treatment of solvation is an extremely complex task to address properly. In principle one should consider models including the interface between the material and a water box thick enough to reproduce a bulk-like behavior far apart from the catalyst. At the same time, the fluxional behavior of the solvent would imply the need for long ab initio molecular dynamics trajectories. To tame the complexity of the problem to make computationally amenable the inclusion of solvation, several approximated schemes have been presented. It is fundamental to remember that any working scheme is not free from limitations and one must try to extract as much chemical information as possible from numbers containing an unavoidable degree of inaccuracy. Here, we decided to adopt the implicit solvation scheme. This approximation does not induce relevant computational extra costs, but neglects direct and directional interactions.

## Supplementary Material



## Data Availability

The data reported in this article can be found in the literature cited and can be asked to the authors upon reasonable request.

## Notes

S1: stability diagrams of 18 SACs considered as a function of pH and voltage; S2: hydrogen evolution reaction and free energy profiles; S3: oxygen evolution reaction and free energy profiles; S4: TM@Au-ene versus TM@Au(111); S5: working equations to compute Gibbs free energies of reaction intermediates; S6: contribution of the solvent to reaction profiles (PDF)
