# Supplementary material for: Single-Atom Catalysts on Goldene
Source: ACS Catal. 2025 Jun 13;15(13):11232–42. doi: 10.1021/acscatal.5c01820 (PMC12235748; doi:10.1021/acscatal.5c01820)
Supplement: Supplementary file 1 [file cs5c01820_si_001.pdf]

Supporting Information

Single-Atom Catalysts on Goldene

Silvia Picello, Elisabetta Inico, Clara Saetta, Giovanni Di Liberto, and Gianfranco Pacchioni*

Department of Materials Science, University of Milano-Bicocca, Via Cozzi 55, 20125 Milano, Italy

* Corresponding author: giovanni.diliberto@unimib.it

Section S1: Stability Diagrams

The stability of SACs against pH and applied voltage was estimated by using the approach proposed in[1]. A thermodynamic cycle is constructed having the following ingredients: the metal binding energy to the support, the energy gain when forming the bulk metal phase from isolated metal atoms and the several possible dissolution pathways in solution from the bulk metal phase. Pourbaix diagrams are constructed where for each pH-E couple the most stable species is reported. The stability of reaction intermediates is calculated by means of the Computational Hydrogen Electrode (CHE). The metal binding energy is taken from DFT simulations. The experimental bulk cohesive energies (E_c) and redox potentials are used. The following redox processes were considered:

V
E_c= 5.31 eV
$V^{2+} + 2e^- \rightarrow V$ $E_{V2+/V}$ = -1.18 V
$V^{3+} + 3e^- \rightarrow V$ $E_{V3+/V}$ = -0.87 V
$VO^+ + 2H^+ + 2e^- \rightarrow V + H_2O$ $E_{VO+/V}$ = -0.57 V
$V_2O_3 + 6H^+ + 6e^- \rightarrow V + 3H_2O$ $E_{V2O3/V}$ = -0.26 V
$V_2O_5 + 10H^+ + 10e^- \rightarrow V + 5H_2O$ $E_{V2O5/V}$ = -0.06 V

Cr
E_c= 4.10 eV
$Cr^{2+} + 2e^- \rightarrow Cr$ $E_{Cr2+/Cr}$ = -0.91 V
$Cr^{3+} + 3e^- \rightarrow Cr$ $E_{Cr3+/Cr}$ = -0.74 V
$Cr(OH)_3 + 3H^+ + 3e^- \rightarrow Cr + 3H_2O$ $E_{Cr(OH)3/Cr}$ = -0.69 V
$HCrO_4^- + 7H^+ + 6e^- \rightarrow Cr + 3H_2O$ $E_{HCrO4-/Cr}$ = +0.31 V

Mn
E_c= 2.92 eV
$Mn^{2+} + 2e^- \rightarrow Mn$ $E_{Mn2+/Mn}$ = -1.19 V
$Mn^{3+} + 3e^- \rightarrow Mn$ $E_{Mn3+/Mn}$ = -0.28 V
$Mn^{4+} + 4e^- \rightarrow Mn$ $E_{Mn4+/Mn}$ = + 0.20 V
$Mn(OH)_2 + 2H^+ + 2e^- \rightarrow Mn + 2H_2O$ $E_{Mn(OH)2/Mn}$ = -0.73 V
$Mn(OH)_3 + 3H^+ + 3e^- \rightarrow Mn + 3H_2O$ $E_{Mn(OH)3/Mn}$ = -0.16 V
$Mn(OH)_4 + 4H^+ + 4e^- \rightarrow Mn + 4H_2O$ $E_{Mn(OH)4/Mn}$ = +0.21 V
$MnO_4^- + 8H^+ + 7e^- \rightarrow Mn + 4H_2O$ $E_{MnO4-/Mn}$ = +0.74 V

Fe

E_c= 4.28 eV
$Fe^{2+} + 2e^- \rightarrow Fe$ $E_{Fe2+/Fe}$ = -0.45 V
$Fe^{3+} + 3e^- \rightarrow Fe$ $E_{Fe3+/Fe}$ = -0.04 V
$Fe(OH)_2 + 2H^+ + 2e^- \rightarrow Fe + 2H_2O$ $E_{Fe(OH)2/Fe}$ = +0.37 V
$Fe(OH)_3 + 3H^+ + 3e^- \rightarrow Fe + 3H_2O$ $E_{Fe(OH)3/Fe}$ = +0.06 V
$FeO_4^{2-} + 8H^+ + 6e^- \rightarrow Fe + 4H_2O$ $E_{FeO42-/Fe}$ = +1.08 V

Co
E_c= 4.39 eV
$Co^{2+} + 2e^- \rightarrow Co$ $E_{Co2+/Co}$ = -0.28 V
$Co^{3+} + 3e^- \rightarrow Co$ $E_{Co3+/Co}$ = +0.45 V
$Co(OH)_2 + 2H^+ + 2e^- \rightarrow Co + 2H_2O$ $E_{Co(OH)2/Co}$ = +0.10 V
$Co(OH)_3 + 3H^+ + 3e^- \rightarrow Co + 3H_2O$ $E_{Co(OH)3/Co}$ = +0.39 V

Ni
E_c= 4.44 eV
$Ni^{2+} + 2e^- \rightarrow Ni$ $E_{Ni2+/Ni}$ = -0.25 V
$Ni(OH)_2 + 2H^+ + 2e^- \rightarrow Ni + 2H_2O$ $E_{Ni(OH)2/Ni}$ = +0.11 V

Cu
E_c= 3.49 eV
$Cu^+ + e^- \rightarrow Cu$ $E_{Cu+/Cu}$ = +0.52 V
$Cu^{2+} + 2e^- \rightarrow Cu$ $E_{Cu2+/Cu}$ = +0.34 V
$Cu(OH)_2 + 2H^+ + 2e^- \rightarrow Cu + 2H_2O$ $E_{Cu(OH)2/Cu}$ = +0.20 V

Nb
E_c= 7.57 eV
$Nb^{3+} + 3e^- \rightarrow Nb$ $E_{Nb3+/Nb}$ = -1.10 V
$NbO + 2H^+ + 2e^- \rightarrow Nb + H_2O$ $E_{NbO/Nb}$ = -0.73 V
$Nb_2O_5 + 10H^+ + 10e^- \rightarrow 2Nb + 5H_2O$ $E_{Nb2O5/Nb}$ = -0.64 V
$NbO_2 + 4H^+ + 4e^- \rightarrow Nb + 2H_2O$ $E_{NbO2/Nb}$ = -0.68 V

Mo
E_c= 6.82 eV
$Mo^{3+} + 3e^- \rightarrow Mo$ $E_{Mo3+/Mo}$ = -0.20 V
$MoO_2 + 4H^+ + 4e^- \rightarrow Mo + 2H_2O$ $E_{MoO2/Mo}$ = -0.15 V
$MoO_3 + 6H^+ + 6e^- \rightarrow Mo + 3H_2O$ $E_{MoO3/Mo}$ = +0.06 V
$MoO_4^{2-} + 8H^+ + 6e^- \rightarrow Mo + 4H_2O$ $E_{MoO42-/Mo}$ = +0.15 V

Ru
E_c= 6.74 eV
$Ru^{2+} + 2e^- \rightarrow Ru$ $E_{Ru2+/Ru}$ = +0.45 V
$Ru^{3+} + 3e^- \rightarrow Ru$ $E_{Ru3+/Ru}$ = +0.38 V
$Ru(OH)_2 + 2H^+ + 2e^- \rightarrow Ru + 2H_2O$ $E_{Ru(OH)2/Ru}$ = +0.68 V
$RuO_4^{2-} + 8H^+ + 6e^- \rightarrow Ru + 4H_2O$ $E_{RuO42-/Ru}$ = +1.19 V

Rh
E_c= 5.75 eV
$Rh^+ + e^- \rightarrow Rh$ $E_{Rh+/Rh}$ = +0.60 V
$Rh^{2+} + 2e^- \rightarrow Rh$ $E_{Rh2+/Rh}$ = +0.60 V
$Rh^{3+} + 3e^- \rightarrow Rh$ $E_{Rh3+/Rh}$ = +0.80 V

$Rh_2O + 2H^+ + 2e^- \rightarrow 2Rh + H_2O$ $E_{Rh2O/Rh}$ = +0.80 V
$Rh_2O_3 + 6H^+ + 6e^- \rightarrow 2Rh + 3H_2O$ $E_{Rh2O3/Rh}$ = +0.75 V
$2RhO_4^{2-} + 16H^+ + 12e^- \rightarrow 2Rh + 8H_2O$ $E_{RhO42-/Rh}$ = +0.54 V

Pd
E_c= 3.39 eV
$Pd^{2+} + 2e^- \rightarrow Pd$ $E_{Pd2+/Pd}$ = 0.92 V
$PdO + 2H^+ + 2e^- \rightarrow Pd + H_2O$ $E_{PdO/Pd}$ = 0.79 V

Ag
E_c= 2.95 eV
$Ag^+ + e^- \rightarrow Ag$ $E_{Ag+/Ag}$ = +0.80 V
$Ag_2O + 2H^+ + 2e^- \rightarrow 2Ag + H_2O$ $E_{Ag2O/Ag}$ = +0.67 V

Ta
E_c= 8.10 eV
$Ta_2O_5 + 10H^+ + 10e^- \rightarrow Ta + 5H_2O$ $E_{Ta2O5/Ta}$ = -0.75 V

W
E_c= 8.90 eV
$WO_2 + 4H^+ + 4e^- \rightarrow W + 2H_2O$ $E_{WO2/W}$ = -0.12 V
$WO_3 + 6H^+ + 6e^- \rightarrow W + 3H_2O$ $E_{WO3/W}$ = -0.09 V
$WO_42- + 8H^+ + 6e^- \rightarrow W + 4H_2O$ $E_{WO42-/W}$ = +0.05 V

Os
E_c= 8.17 eV
$Os(OH)_4 + 4H^+ + 4e^- \rightarrow Os + 2H_2O$ $E_{Os(OH)4/Os}$ = -0.12 V
$OsO_4^{2-} + 8H^+ + 6e^- \rightarrow Os + 4H_2O$ $E_{OsO42-/Os}$ = +0.99 V
$OsO_5^{2-} + 10H^+ + 8e^- \rightarrow Os + 5H_2O$ $E_{OsO52-/Os}$ = +0.82 V
$OsO_4 + 8H^+ + 8e^- \rightarrow Os + 4H_2O$ $E_{OsO4/Os}$ = +0.84 V

Ir
E_c= 6.94 eV
$Ir^{3+} + 3e^- \rightarrow Ir$ $E_{Ir3+/Ir}$ = +1.16 V
$IrO + 2H^+ + 2e^- \rightarrow Ir + H_2O$ $E_{IrO/Ir}$ = +0.87 V
$IrO^2 + 4H^+ + 4e^- \rightarrow Ir + 2H_2O$ $E_{IrO2/Ir}$ = +0.93 V
$IrO_4^{2-} + 8H^+ + 6e^- \rightarrow Ir + 4H_2O$ $E_{IrO42-/Ir}$ = +0.15 V

Pt
E_c= 5.84 eV
$Pt^{2+} + 2e^- \rightarrow Pt$ $E_{Pt2+/Pt}$ = 1.19 V

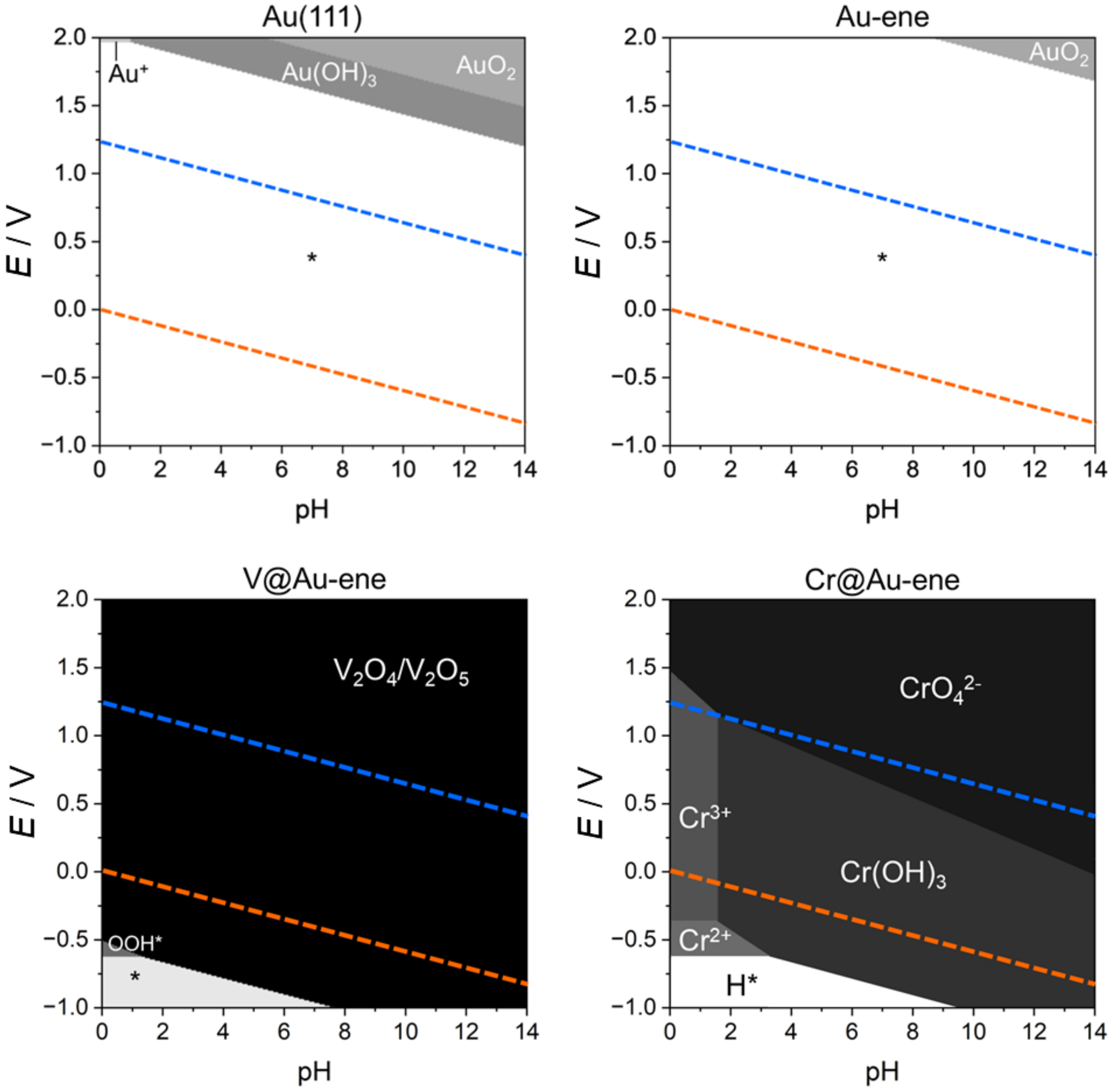

Figure S1. *Stability diagrams as a function of (pH, E). Top: Au(111) and Au-ene (goldene); bottom: V@Au-ene and Cr@Au-ene.*

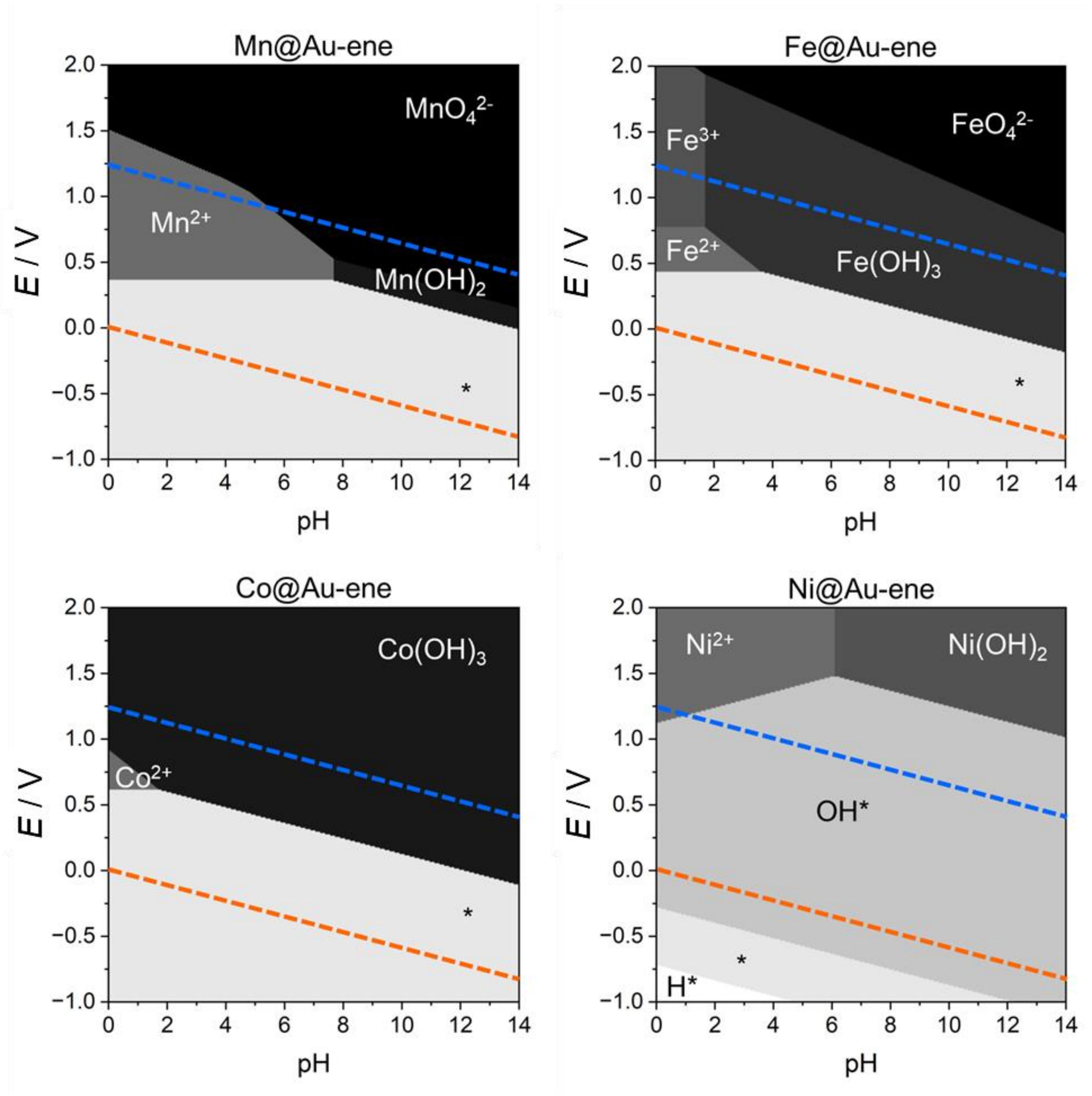

Figure S2. *Stability diagrams as a function of (pH, E) of TM@Au-ene (Mn, Fe, Co and Ni).*

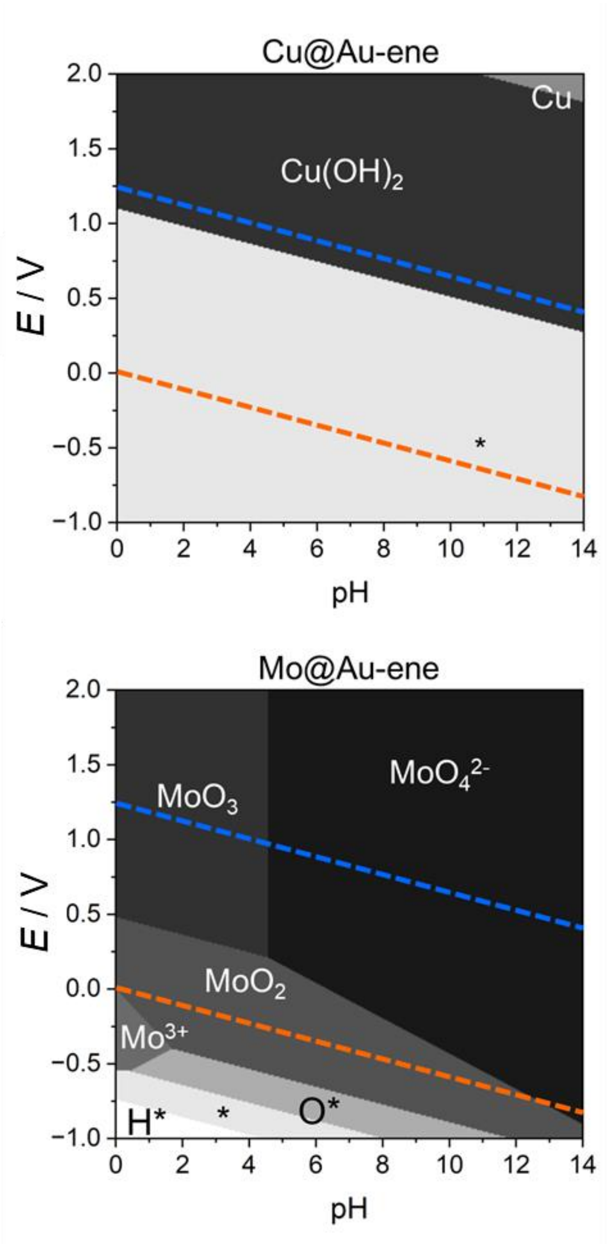

Figure S3. *Stability diagrams as a function of (pH, E) of TM@Au-ene (Cu, Nb, Mo and Ru).*

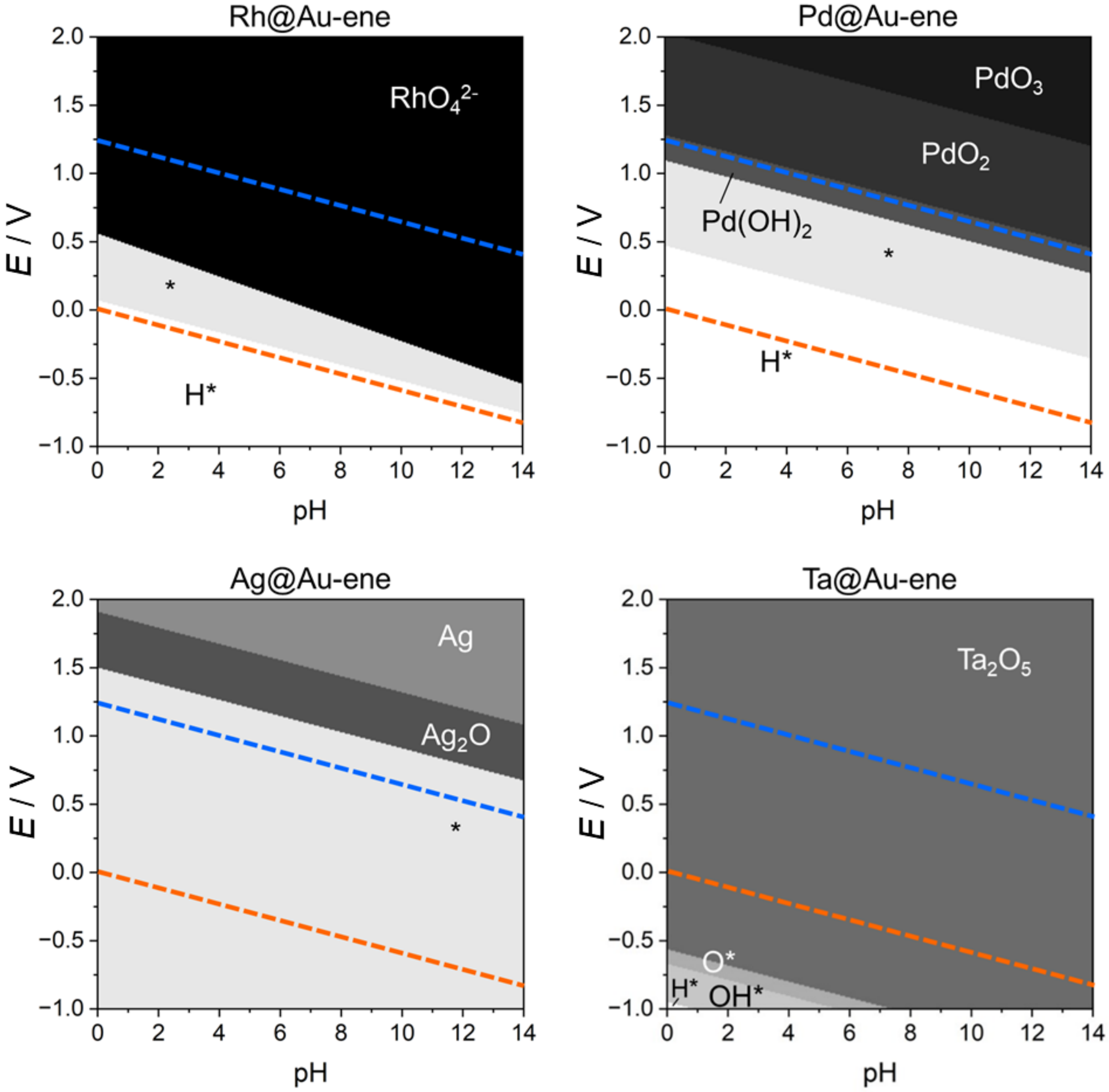

Figure S4. *Stability diagrams as a function of (pH, E) of TM@Au-ene (Rh, Pd, Ag and Ta).*

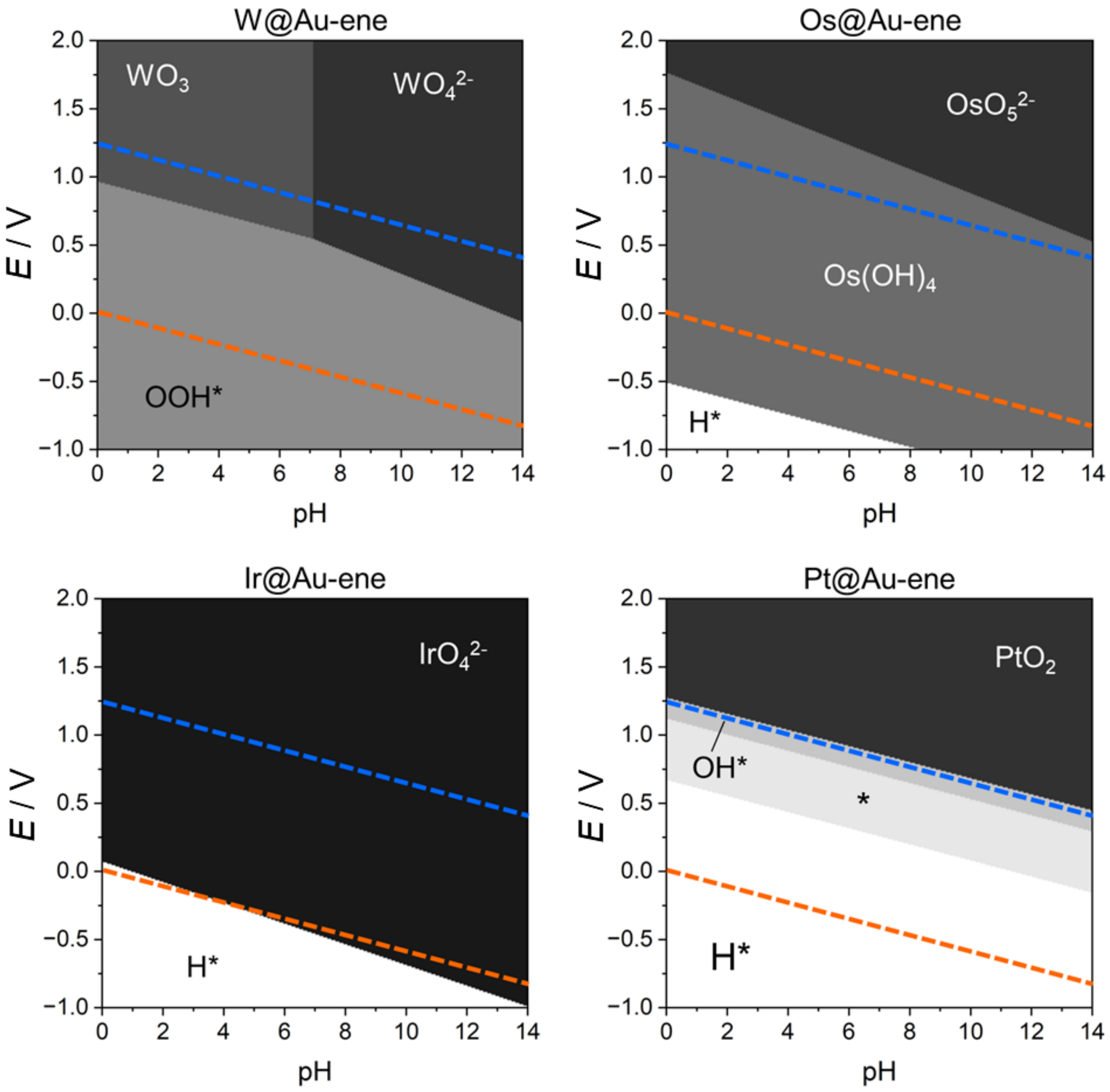

Figure S5. *Stability diagrams as a function of (pH, E) of TM@Au-ene (W, Os, Ir and Pt).*

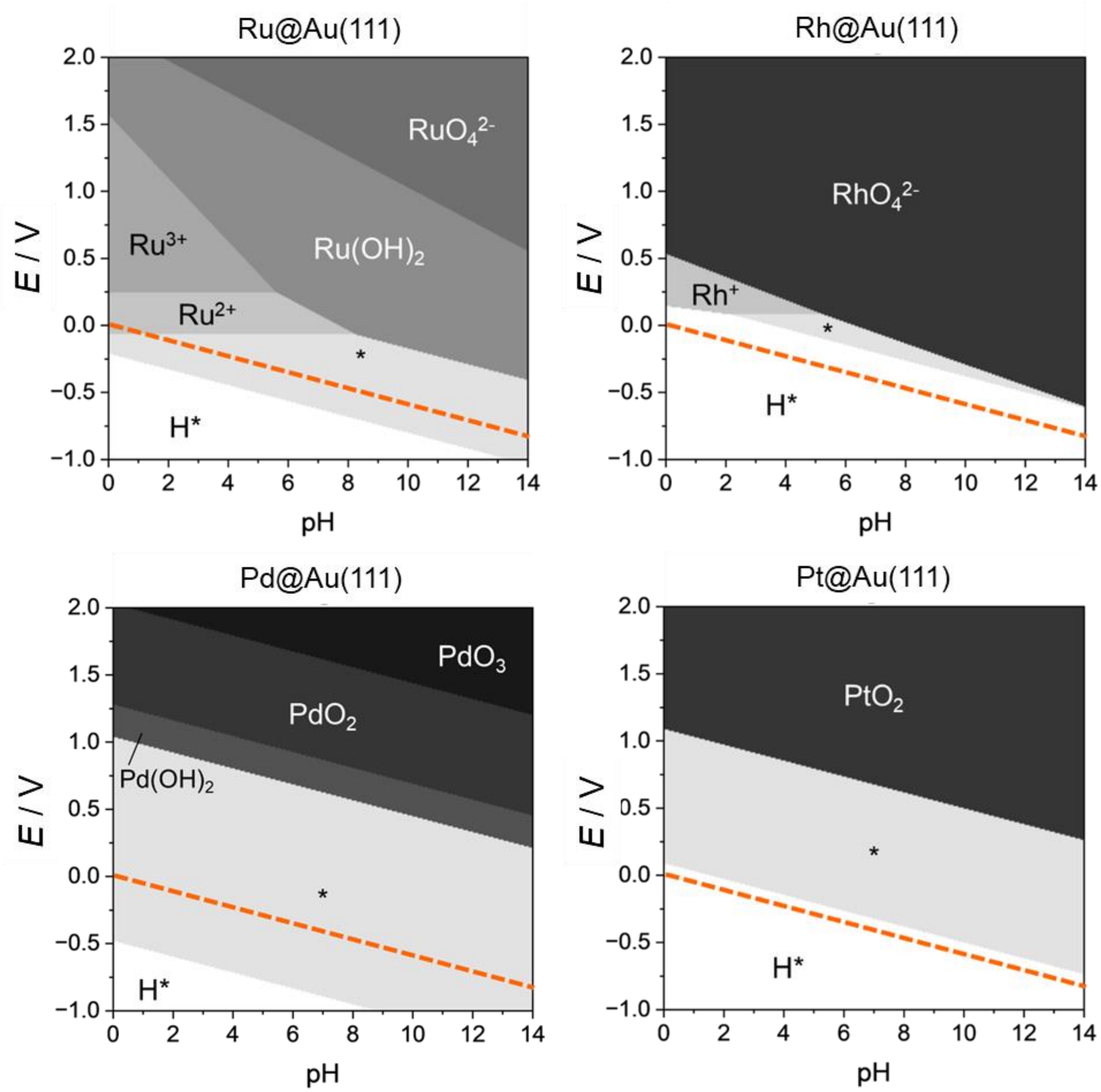

Figure S6. *Stability diagrams as a function of (pH, E) of TM@Au(111) (Ru, Rh, Pd and Pt).*

Section S2: Hydrogen Evolution

Table S1*. Formation free energy of H* adducts and shortest distances,*

TM@Au-ene	ΔG_{H*} / eV	d_{TM-H} / Å
V	1.42	1.65
Cr	-0.74	1.62
Mn	1.49	1.61
Fe	1.40	1.53
Co	1.61	1.52
Ni	0.72	1.41
Cu	1.10	1.45
Nb	-0.08	1.74
Mo	0.74	1.66
Ru	0.34	1.59
Rh	-0.07	1.50
Pd	-0.47	1.47
Ag	1.28	1.59
Ta	-0.42	1.75
W	-0.10	1.68
Os	0.10	1.59
Ir	-0.51	1.52
Pt	-0.67	1.50
Au	0.75	1.53

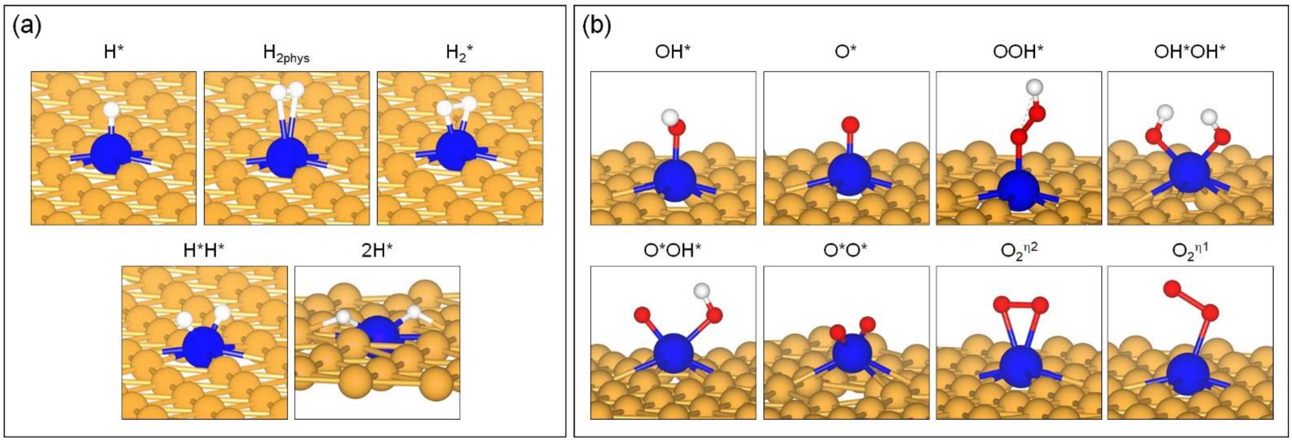

Figure S7*. (a) Structures of HER intermediates; (b) Structures of OER intermediates.*

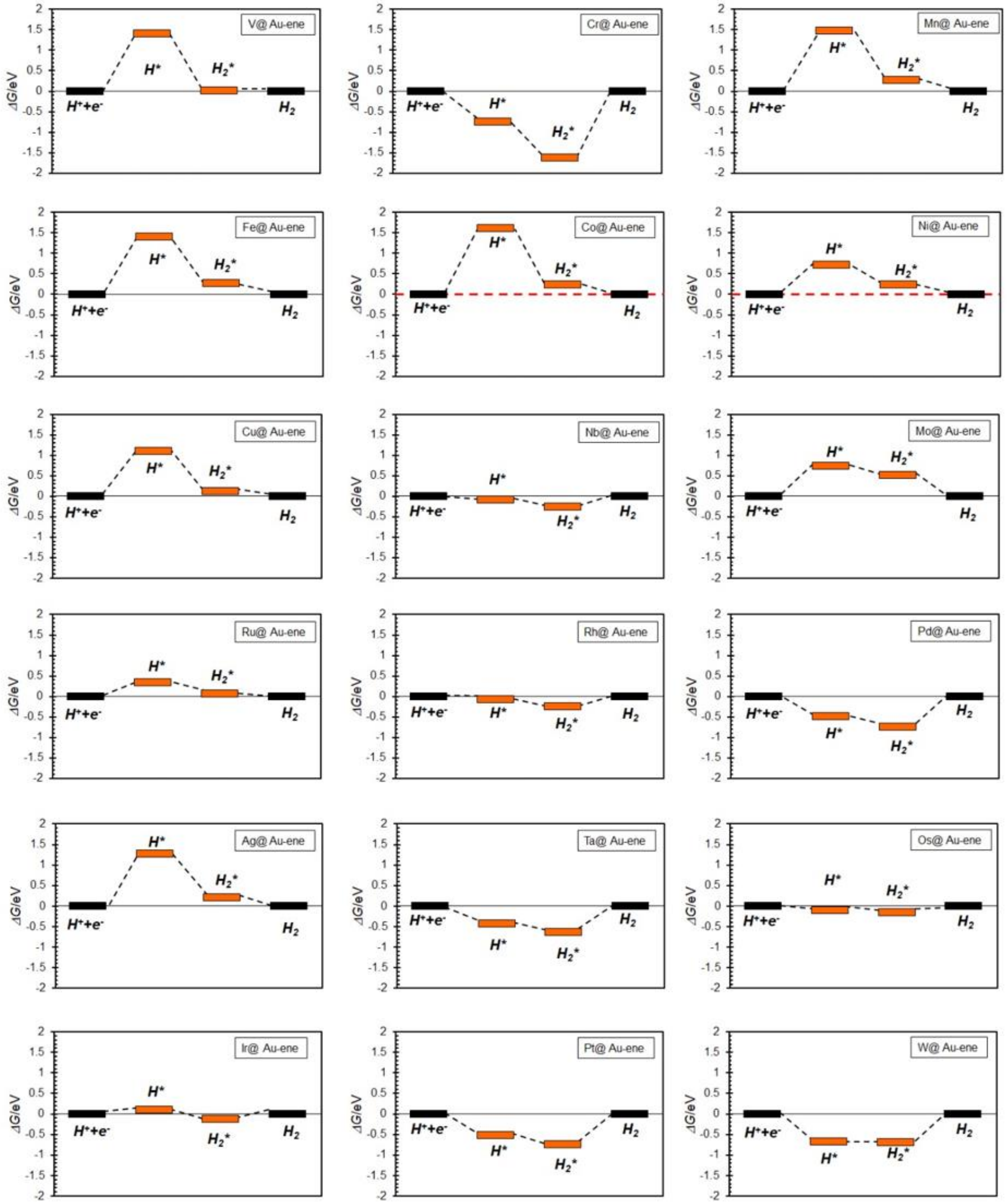

Figure S8. *Free energy profiles for HER on TM@Au-ene.*

Section S3: Oxygen Evolution

Table S2. *O-O distances in O_2-complexes on TM@Au-ene.*

**TM@Au-ene**	$d_{O\text{-}O}$ **/ Å**
V	1.45
Cr	2.67
Mn	2.87
Fe	1.32
Co	3.17
Ni	1.79
Cu	3.50
Nb	2.74
Mo	1.42
Ru	1.38
Rh	1.32
Pd	1.30
Ag	2.64
Ta	2.77
W	2.27
Os	1.41
Ir	1.34
Pt	1.31
Au	1.24

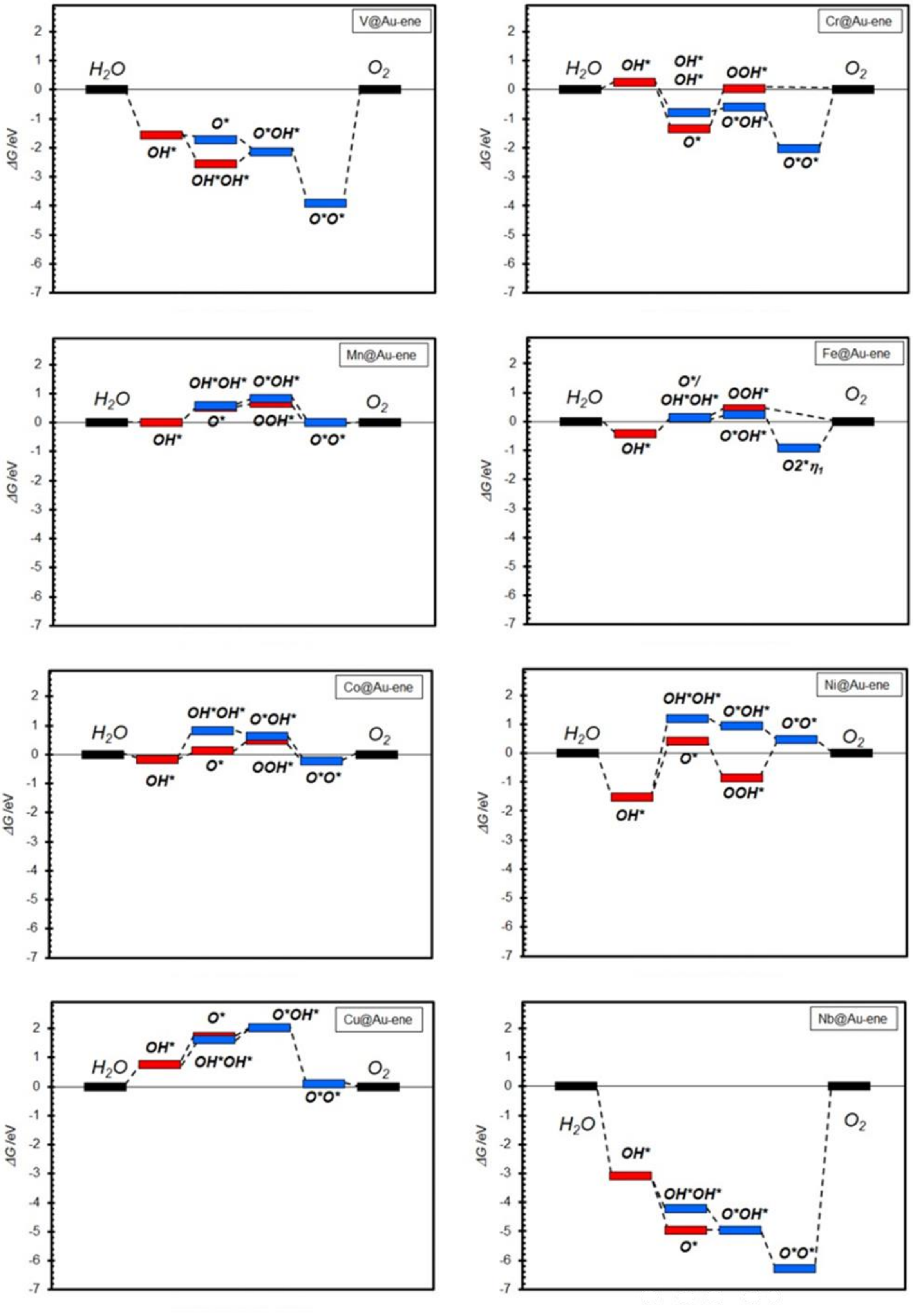

Figure S9. *Free energy profiles for OER on TM@Au-ene (Conventional pathway in red, Unconventional in blue). TM = V, Cr, Mn, Fe, Co, Ni, Cu, Nb.*

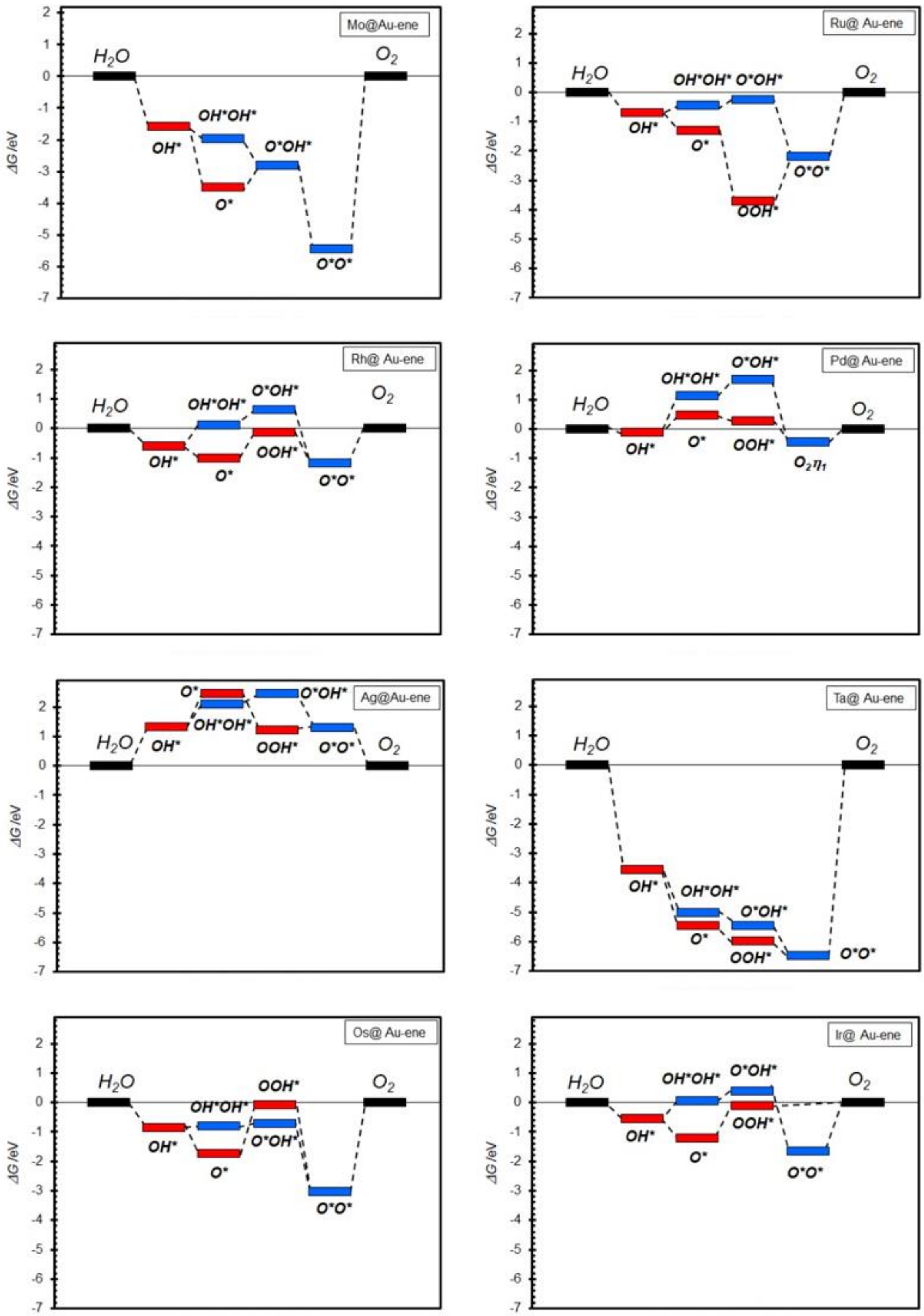

Figure S10. *Free energy profiles for OER on TM@Au-ene (Conventional pathway in red, Unconventional in blue). TM = Mo, Ru, Rh, Pd, Ag, Ta, Os, Ir.*

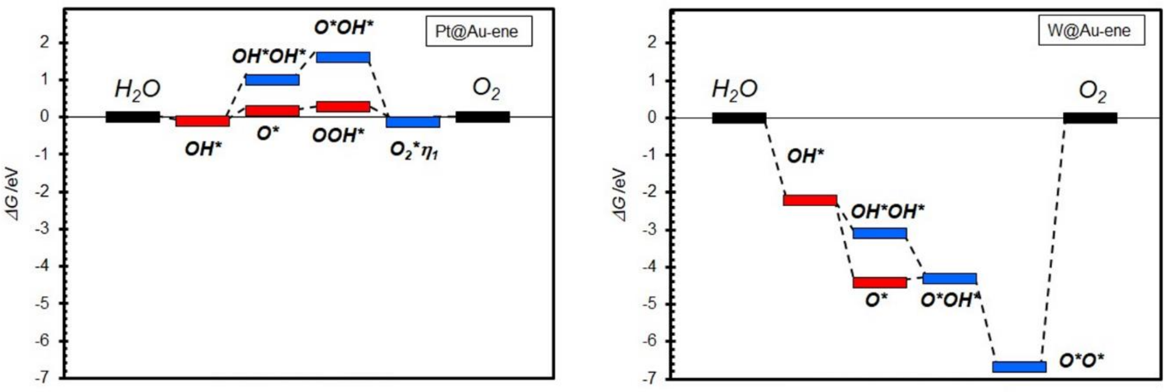

Figure S11. *Free energy profiles for OER on TM@Au-ene (Conventional pathway in red, Unconventional in blue). TM = Pt, W.*

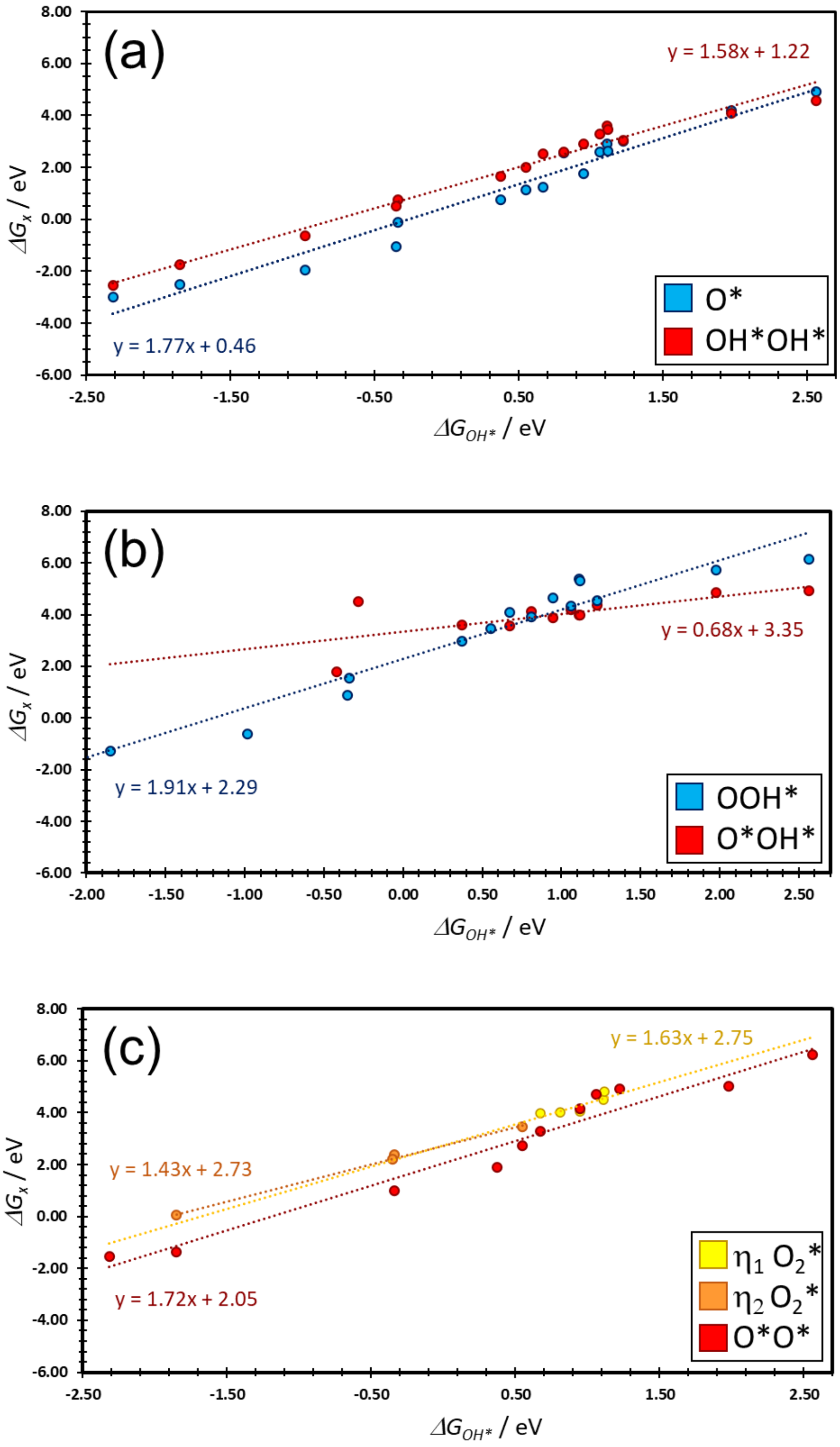

***Figure S12**. Scaling relations for OER, comparing competitive intermediates. (a) O* and OH*OH*; (b) OOH* and O*OH*; (c) all possible oxygen complexes.*

Section S4: TM@Au-ene versus TM@Au(111)

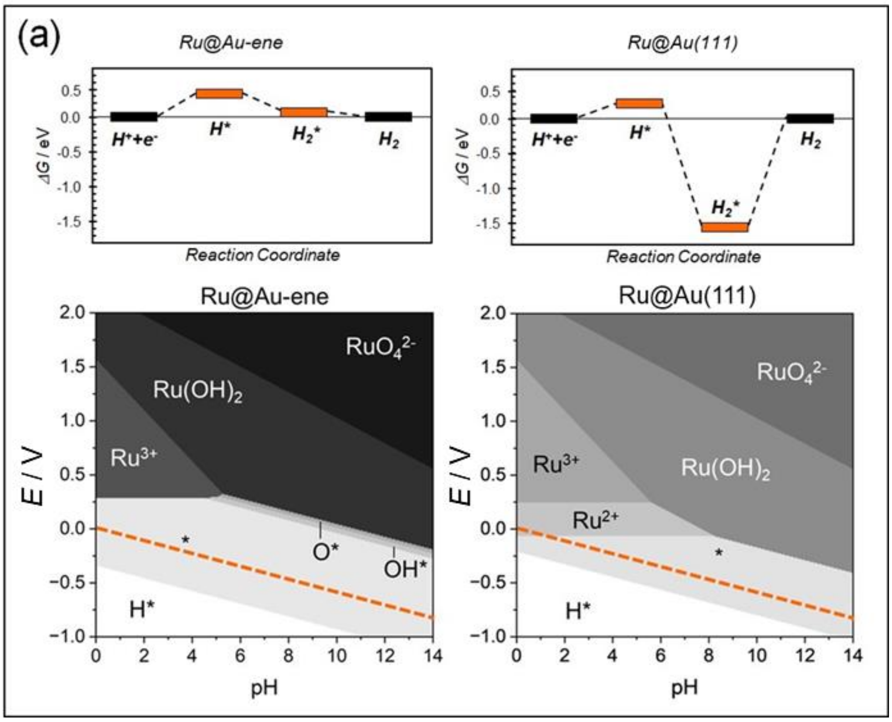

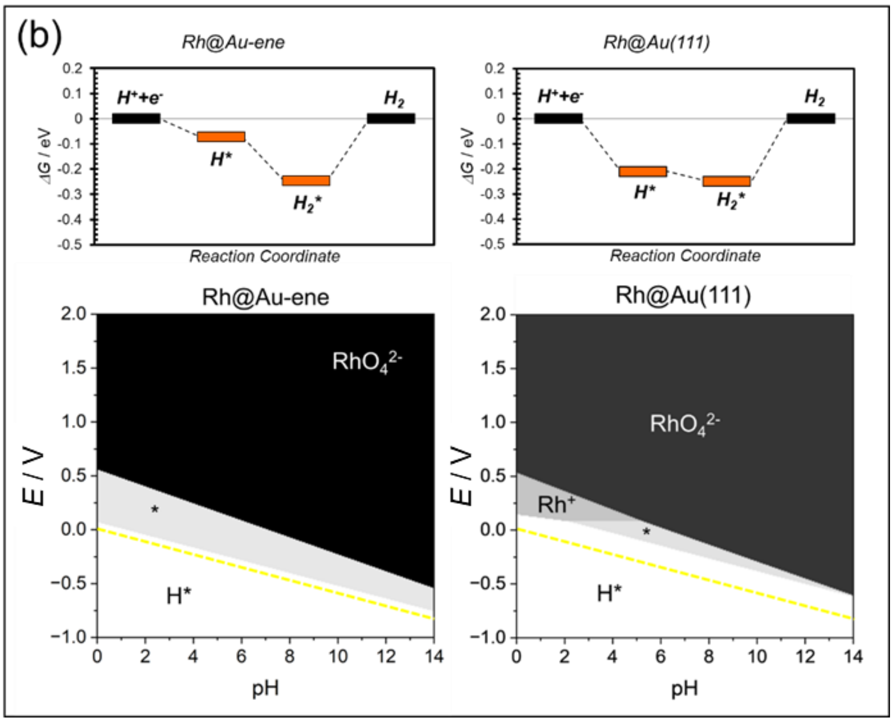

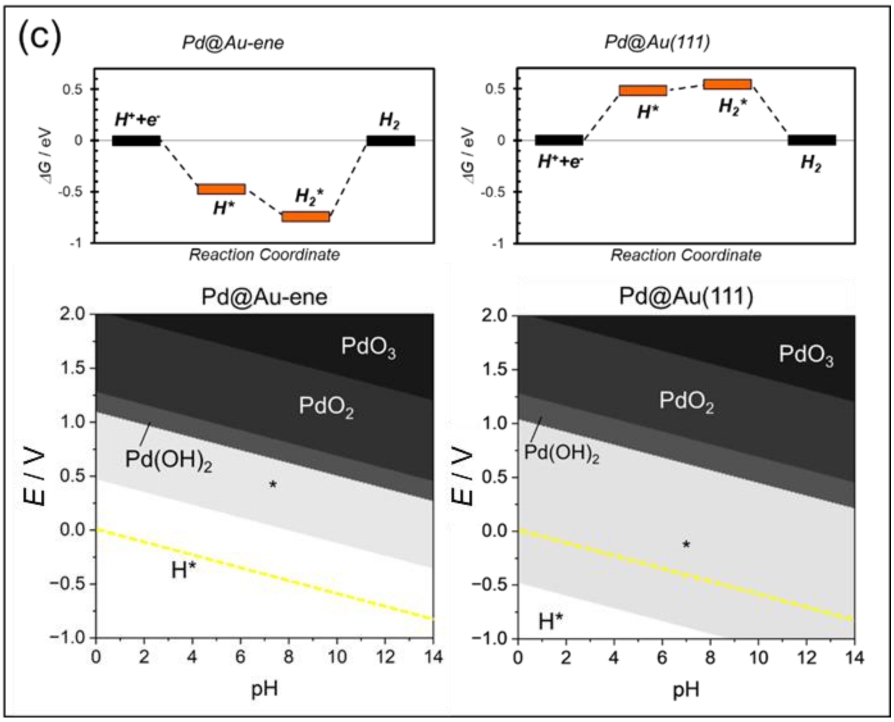

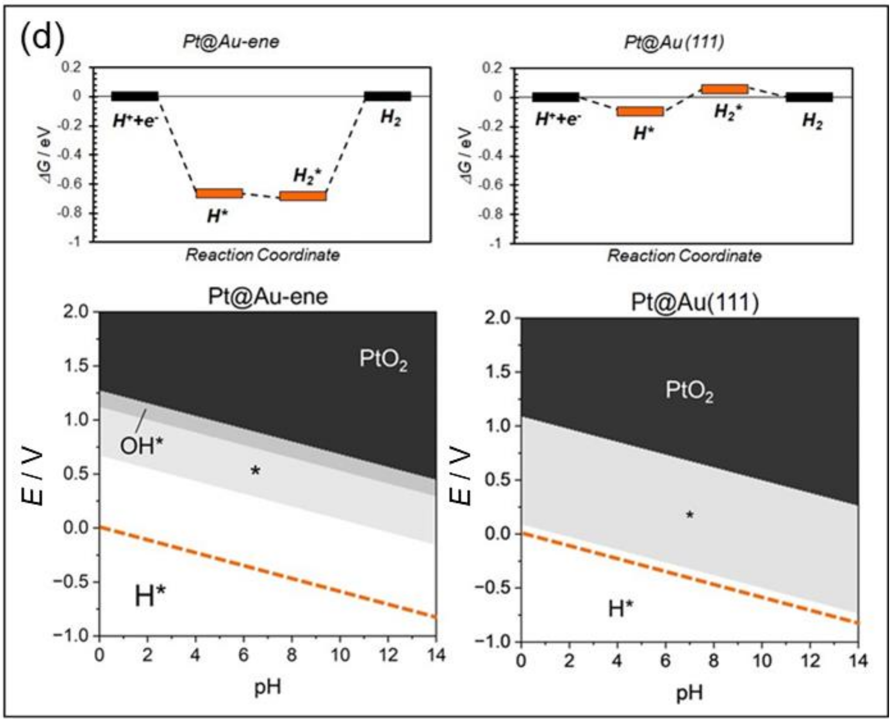

Figure S13. *Comparison of reactivity (top panel) and stability (bottom panel) of TM@Au-ene and TM@Au(111). (a) Ru; (b) Rh; (c) Pd; (d) Pt.*

***Table S3**. Formation free energies and structural properties of HER intermediates on TM@Au-ene and TM@Au(111).*

	TM	ΔG_{H^*} / eV	ΔG_{H2^*} / eV	***distances TM-H*** **/ Å | in H***	***distances TM-H*** **/ Å | in H_2***	***distances H-H*** **/ Å**
Au-ene	Rh	-0.07	-0.25	1.50	1.54	1.83
	Ru	0.34	0.07	1.59	1.59	1.05
	Pd	-0.47	-0.74	1.47	1.61	2.38
	Pt	-0.67	-0.68	1.50	1.59	2.37
Au(111)	Rh	-0.21	-0.25	1.54	1.65	0.93
	Ru	0.21	-0.21	1.58	1.62	2.31
	Pd	0.48	0.30	1.52	1.75	2.81
	Pt	-0.09	0.06	1.54	1.62	2.30

Section S5: Working Equations

The adsorption of energies of adsorbates were calculated according to the following equations:

$\Delta E_{H^*} = E_{H^*} - ½ E_{H2} - E_*$ *Eq. S1*

$\Delta E_{H2\text{-complex}} = E_{H2^*} - E_{H2} - E_*$ *Eq. S2*

$\Delta E_{OH^*} = E_{OH^*} + ½ E_{H2} - E_{H2O} - E_*$ *Eq. S3*

$\Delta E_{O^*} = E_{O^*} + E_{H2} - E_{H2O} - E_*$ *Eq. S4*

$\Delta E_{OOH^*} = E_{OOH^*} + 3/2\, E_{H2} - 2\, E_{H2O} - E_*$ *Eq. S5*

$\Delta E_{OH^*OH^*} = E_{OH^*OH^*} + E_{H2} - 2\, E_{H2O} - E_*$ *Eq. S6*

$\Delta E_{O^*OH^*} = E_{O^*OH^*} + 3/2\, E_{H2} - 2\, E_{H2O} - E_*$ *Eq. S7*

$\Delta E_{O2\text{-complex}} = E_{O2\text{-complex}} + 2\, E_{H2} - 2\, E_{H2O} - E_*$ *Eq. S8*

where E_x is the energy of the catalyst with adsorbed x species and O_2. E_{H2}, E_{H2O} and E_* are the energies of the isolated hydrogen molecule, water molecule and catalyst, respectively. The Gibbs free energy was estimated by including the thermodynamics corrections. The entropy of gas phase H_2 and H_2O was taken from international tables (see Table S1). For the adsorbates we calculated entropies and Zero-Point Energy corrections in the framework of the harmonic fashion. The value of *TS* and *ZPE* of the same adsorbate on different metals is always constant with a variation of 0.01 eV at maximum.

Table S4. *Entropic contribution and Zero-Point Energy values of molecular species and adsorbates.*

**Species**	***TS* / eV**	***ZPE* / eV**
H_2	0.41	0.27
H_2O	0.67	0.56
H*	0.02	0.14
H_2-complex	0.11	0.20
OH*	0.12	0.32
O*	0.05	0.05
OH*OH*	0.50	0.66
OOH*	0.32	0.42
O*OH*	0.33	0.39
η_1O_2*	0.22	0.08
η_2O_2*	0.22	0.08
O*O*	0.19	0.11

Section S6: Solvation contribution

Implicit solvation was used via the VASPsol tool implemented in VASP, where the effect of the solvent is approximated using a continuum dielectric approach, with water represented by a homogeneous medium characterized by a dielectric constant (ε = 78.4).

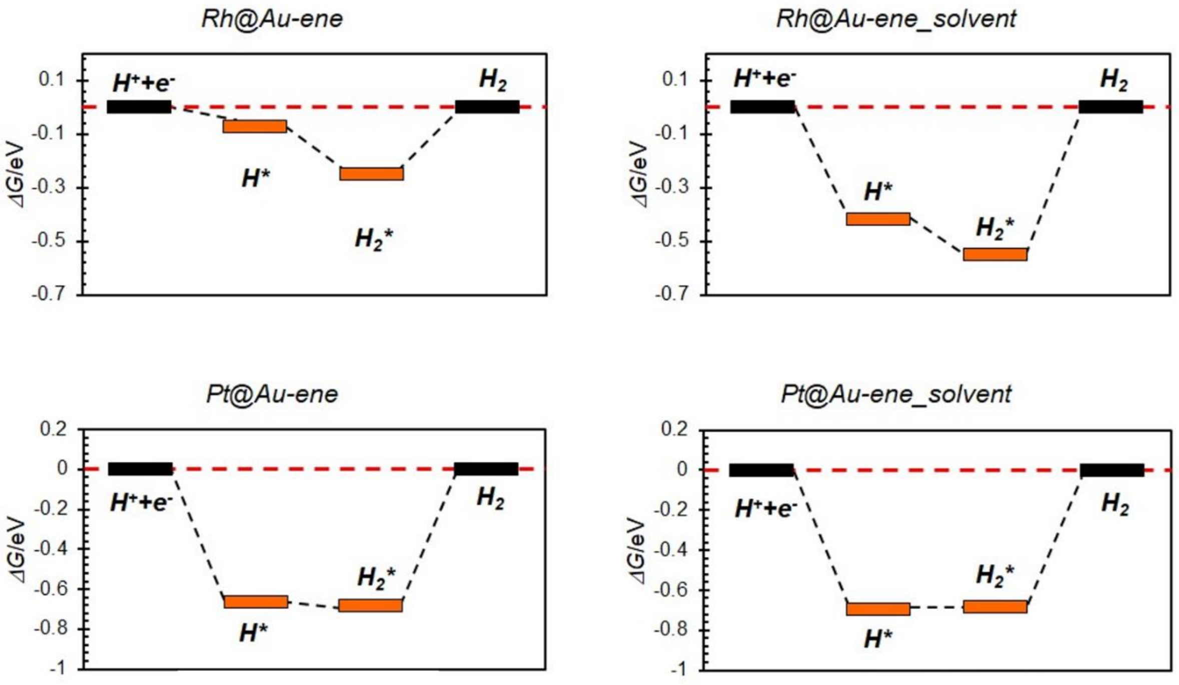

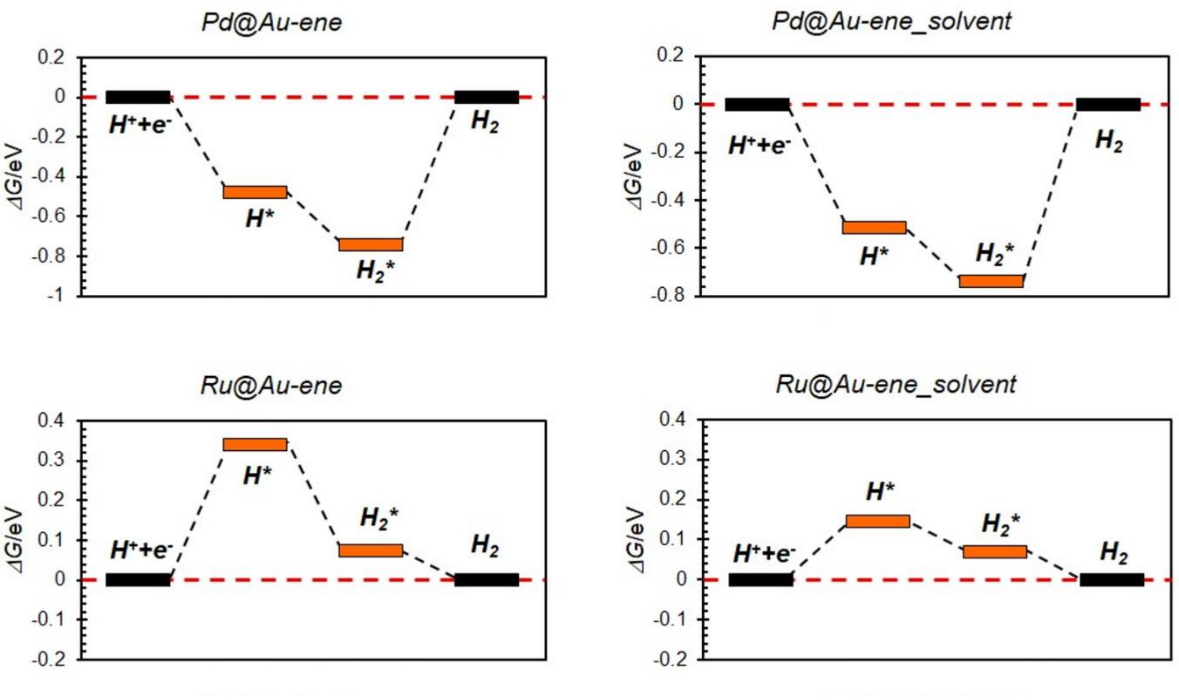

Figure S14. *HER free energy diagrams for selected cases of TM@Au-ene. Left: reaction in vacuum; Right: reaction with implicit solvation.*

Table S5. *Formation free energies and structural properties of HER intermediate without (dry) and with (solvent) implicit solvation.*

	TM@Au-ene	ΔG_{H^*} / eV	$\Delta G_{H_2^*}$ / eV	distances TM-H / Å | in H*	distances TM-H / Å | in H_2*	distances H-H / Å	$\Delta\Delta G_{H^*}$ / eV	$\Delta\Delta G_{H_2^*}$ / eV
dry	Rh	-0.07	-0.25	1.5	1.54	1.83		
	Ru	0.34	0.07	1.59	1.59	1.05		
	Pd	-0.47	-0.74	1.47	1.61	2.38		
	Pt	-0.67	-0.68	1.5	1.59	2.37		
solvent	Rh	-0.42	-0.55	1.5	1.54	1.83	-0.35	-0.3
	Ru	0.15	-0.07	1.56	1.59	1.05	-0.19	-0.14
	Pd	-0.52	-0.74	1.47	1.61	2.38	-0.05	0
	Pt	-0.7	-0.68	1.5	1.59	2.37	-0.03	0

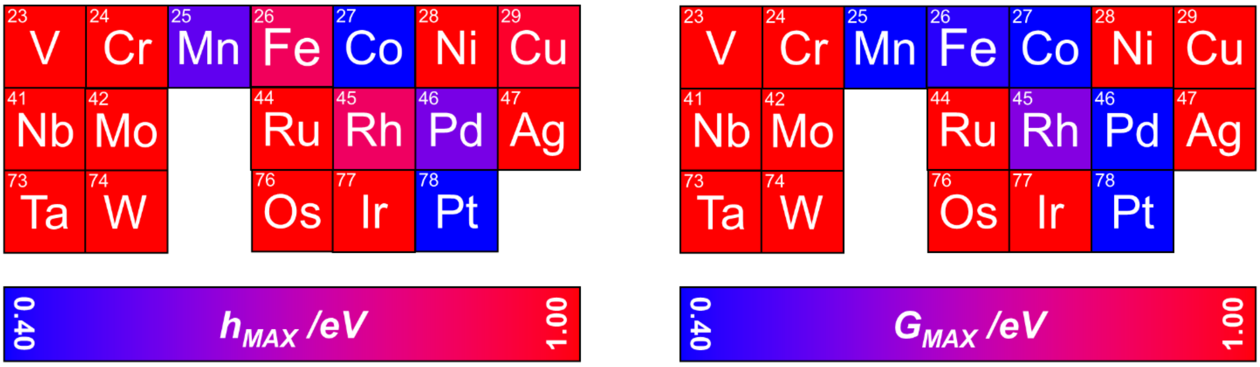

Figure S15. *Activity of the TM atoms considered in this work. On the left the h_{MAX} value is represented, on the right one can see G_{MAX}. The reactivity of each atom is reported with a color scale, depending on the extent of the barrier (blue, h_{MAX} or G_{MAX} < 0.4 eV, red, h_{MAX} or G_{MAX} > 1.00 eV).*

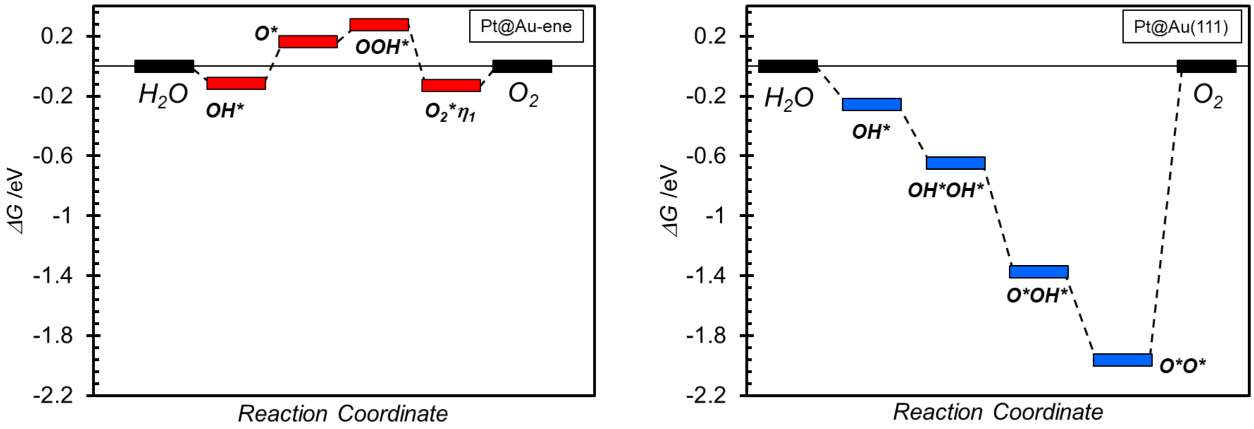

Figure S16. *Oxygen Evolution Reaction pathway happening on Au-ene (on the left, in red) and on Au(111) (on the right, in blue); both calculated at an overpotential of 1.23V.*

References

(1) Di Liberto, G.; Giordano, L.; Pacchioni, G. Predicting the Stability of Single-Atom Catalysts in Electrochemical Reactions. *ACS Catal* **2024**, *14* (1), 45–55. https://doi.org/10.1021/acscatal.3c04801.
